# Supplementary material for: A cytochrome P450 CYP87A4 imparts sterol side-chain cleavage in digoxin biosynthesis
Source: Nat Commun. 2023 Jul 8;14:4042. doi: 10.1038/s41467-023-39719-4 (PMC10329713; doi:10.1038/s41467-023-39719-4)
Supplement: Supplementary file 1 — Supplementary Information [file 41467_2023_39719_MOESM1_ESM.pdf]

A Cytochrome P450 CYP87A4 Imparts Sterol Side-Chain Cleavage in Digoxin Biosynthesis

Emily Carroll[1†], Baradwaj Ravi Gopal[1†], Indu Raghavan[1], Minakshi Mukherjee[1], Zhen Q. Wang[1*]

1. Department of Biological Sciences, University at Buffalo, the State University of New York, Buffalo, New York, NY14260, United States

[†]These authors contributed equally to this work.

**Supplementary Figures**	**Page**
Supplementary Figure 1. Statistical analysis of the assembled transcriptome	3
Supplementary Figure 2. Functional classification of the unigenes from the transcriptome	4
Supplementary Figure 3. KEGG pathway annotation	5
Supplementary Figure 4. PKs, TFs, TRs in the D. lanata transcriptome	6
Supplementary Figure 5. GC/MS analysis of steroids in the leaves of D. lanata	7
Supplementary Figure 6. Differential expression of terpenoid biosynthetic pathways in leaves vs. roots	8
Supplementary Figure 7. Phylogenetic tree with D. lanata and Arabidopsis thaliana cytochrome P450s	9
Supplementary Figure 8. Differentially expressed cytochrome P450s in the leaves and roots of D. lanata	10
Supplementary Figure 9. MS spectra of pathway intermediates in tobacco transiently expressing digoxin biosynthetic pathway genes	11
Supplementary Figure 10. Identifying putative digoxin pathway intermediates in D. lanata	12
Supplementary Figure 11. GC/MS analysis of sterol contents in engineered yeast strains	13
Supplementary Figure 12. Preliminary experiment with campesterol-producing yeast expressing DlCYP87A4	14
Supplementary Figure 13. Multi-sequence alignment of DlCYP87A4 with mammalian CYP11A1 and other plant CYP87As	15
Supplementary Figure 14. The campesterol-producing yeast strain expressing various Digitalis lanata CYP87As	17
Supplementary Figure 15. Metadata from Alphafold2 modeling of DlCYP87A4	18

Supplementary Tables

Supplementary Table 1. Top 20 simple sequence repeats (SSRs) in a total of 22,549 D. lanata SSRs 19

Supplementary Table 2. DlCYP87A4 with closely related cytochrome P450s in various plant species 20

Supplementary Table 3. Primers used in this study 21

Supplementary Table 4. Constructs used in this study 22

Supplementary Table 5. Yeast strains used in this study 24

Supplementary Methods

De novo assembly of transcriptome 25

Transcriptome annotation and functional classification 25

Identification of differentially expressed transcripts 26

Identification of transcription factor and protein kinase families 26

Identification of simple sequence repeats (SSRs) 26

BUSCO analysis 27

Gene isolation and cloning 27

GC/MS for cholesterol and phytosterol quantification 27

Real-time polymerase chain reaction (RT-PCR) 28

Phylogenetic analysis 28

Supplementary Notes

Supplementary Note 1. Transcriptome sequencing and its de novo assembly 29

Supplementary Note 2. Transcriptome annotation 29

Supplementary Note 3. Gene ontology (GO) classification 30

Supplementary Note 4. Functional characterization using KEGG 30

Supplementary Note 5. Protein kinase, transcription regulator, and transcription factor families 30

Supplementary Note 6. Simple sequence repeats (SSRs) 31

Supplementary References 32

Supplementary Figures

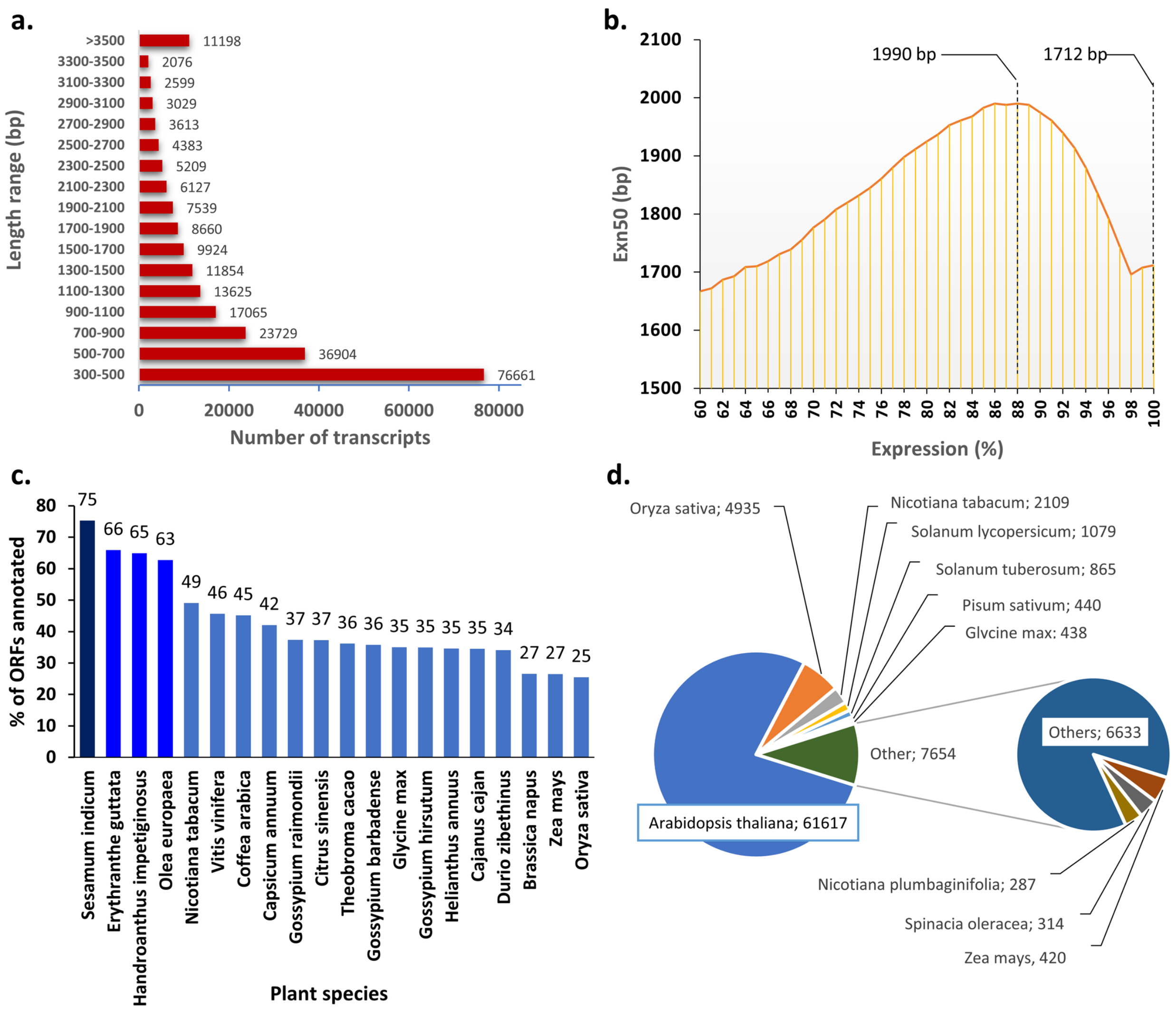

Supplementary Figure 1. Statistical analysis of the assembled transcriptome**. a.** Transcriptome assembly data showing the size distribution of transcripts (N50 = 1,712 bp). **b.** Expression-dependent N50 (ExN50) calculated as the N50 of the top X% of the transcript expression levels (Ex). ExN50 of 1,990 bp at the top 88% expression level and traditional N50 of 1,712 bp at 100% expression level are marked. **c.** Percentage of ORFs annotated by various plant species using BLASTx of the NCBI Non-redundant protein database. **d.** Number of ORFs annotated by various plant species using BLASTx of the Swiss-Prot non-redundant protein database.

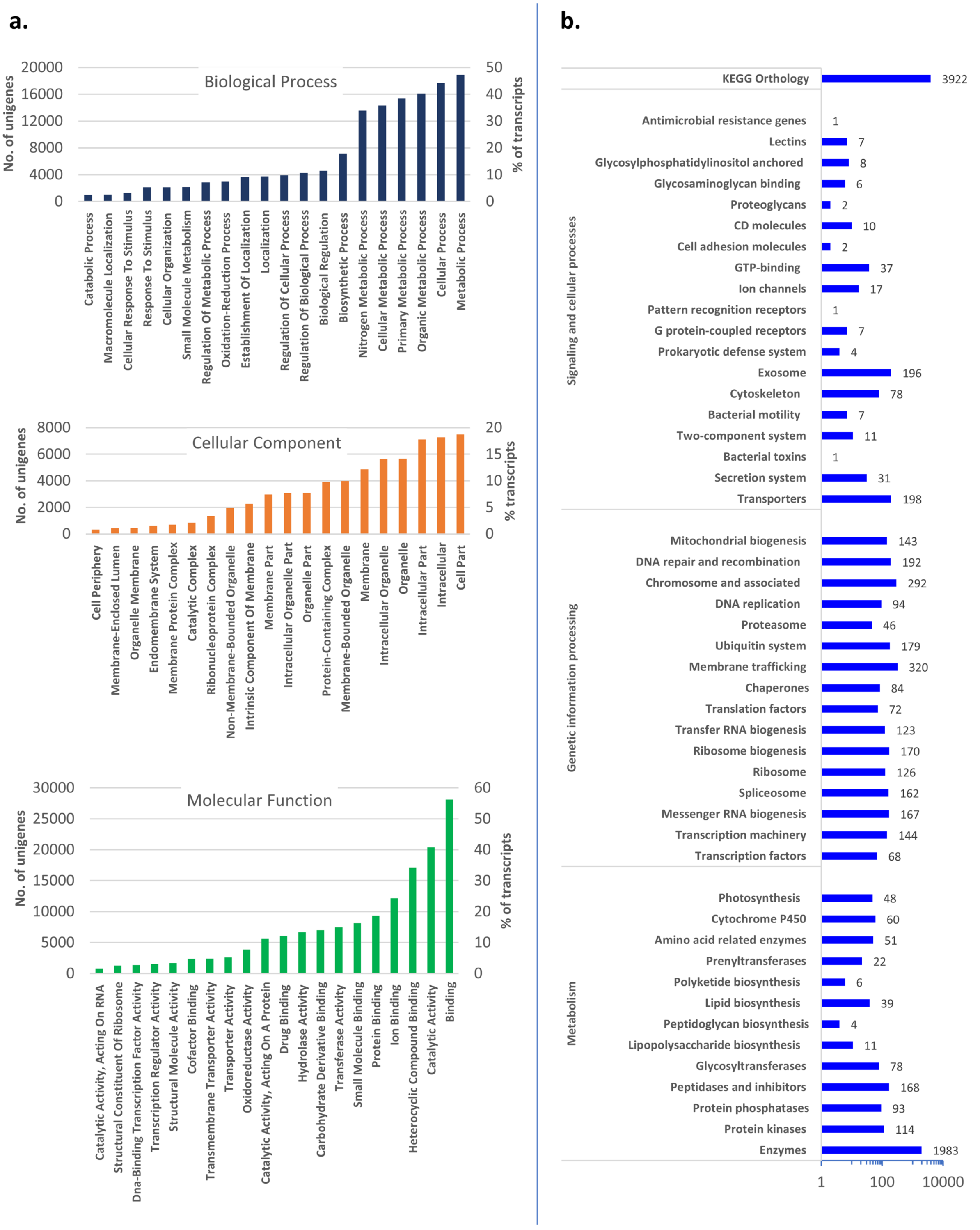

Supplementary Figure 2. Functional classification of the unigenes from the transcriptome. **a.** Unigenes were plotted as numbers and percentages of unigenes matching various GO categories. **b.** KEGG mapping against BRITE hierarchies of unigenes classified into various biological processes. The numbers are various KOs (KEGG orthologs) represented by unigenes.

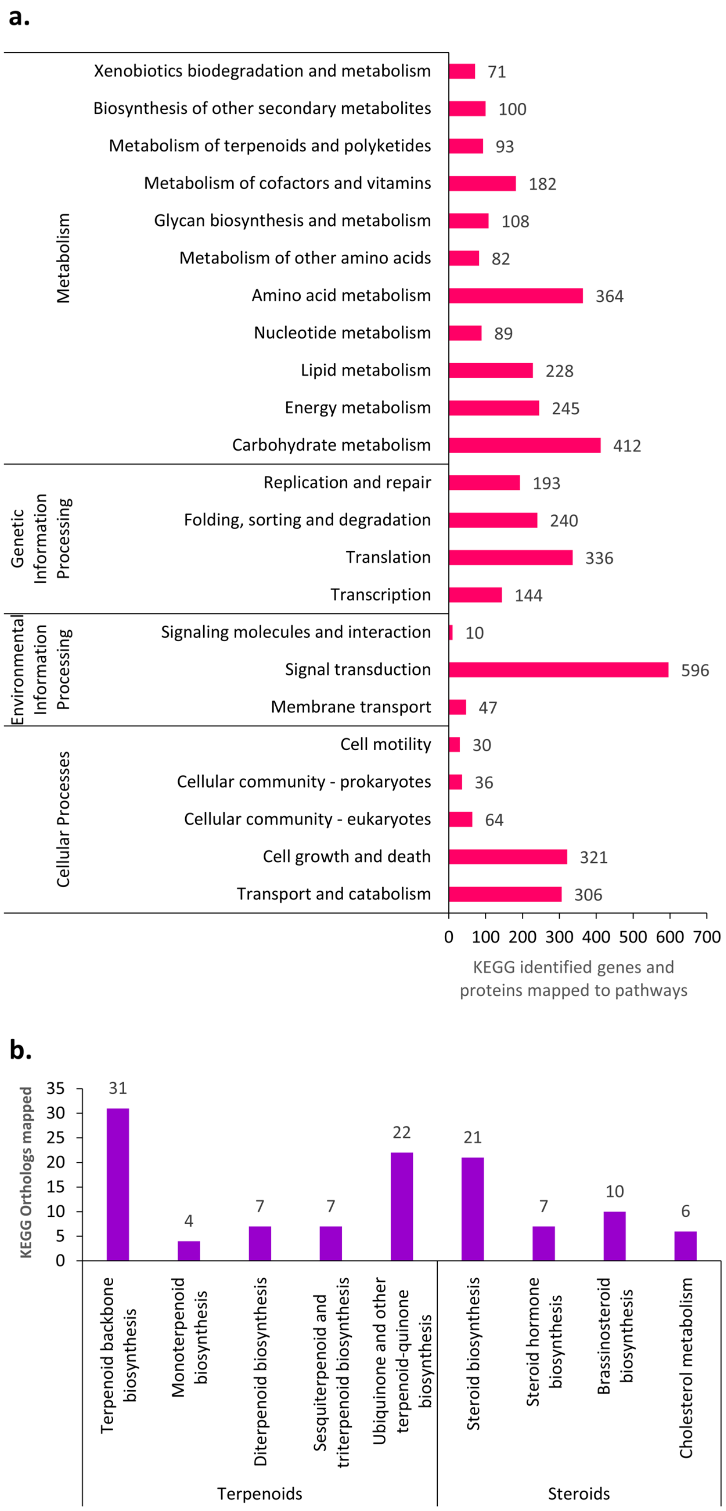

Supplementary Figure 3. KEGG pathway annotation. **a.** KEGG pathway classification map. Unigenes were allocated biological pathways based on their KO (KEGG ortholog) annotations. The values represent the number of KOs answered by unigenes. **b.** KEGG analysis showing that KEGG orthologs (KO) involving in terpenoid and steroid pathways are represented by unigenes in this study.

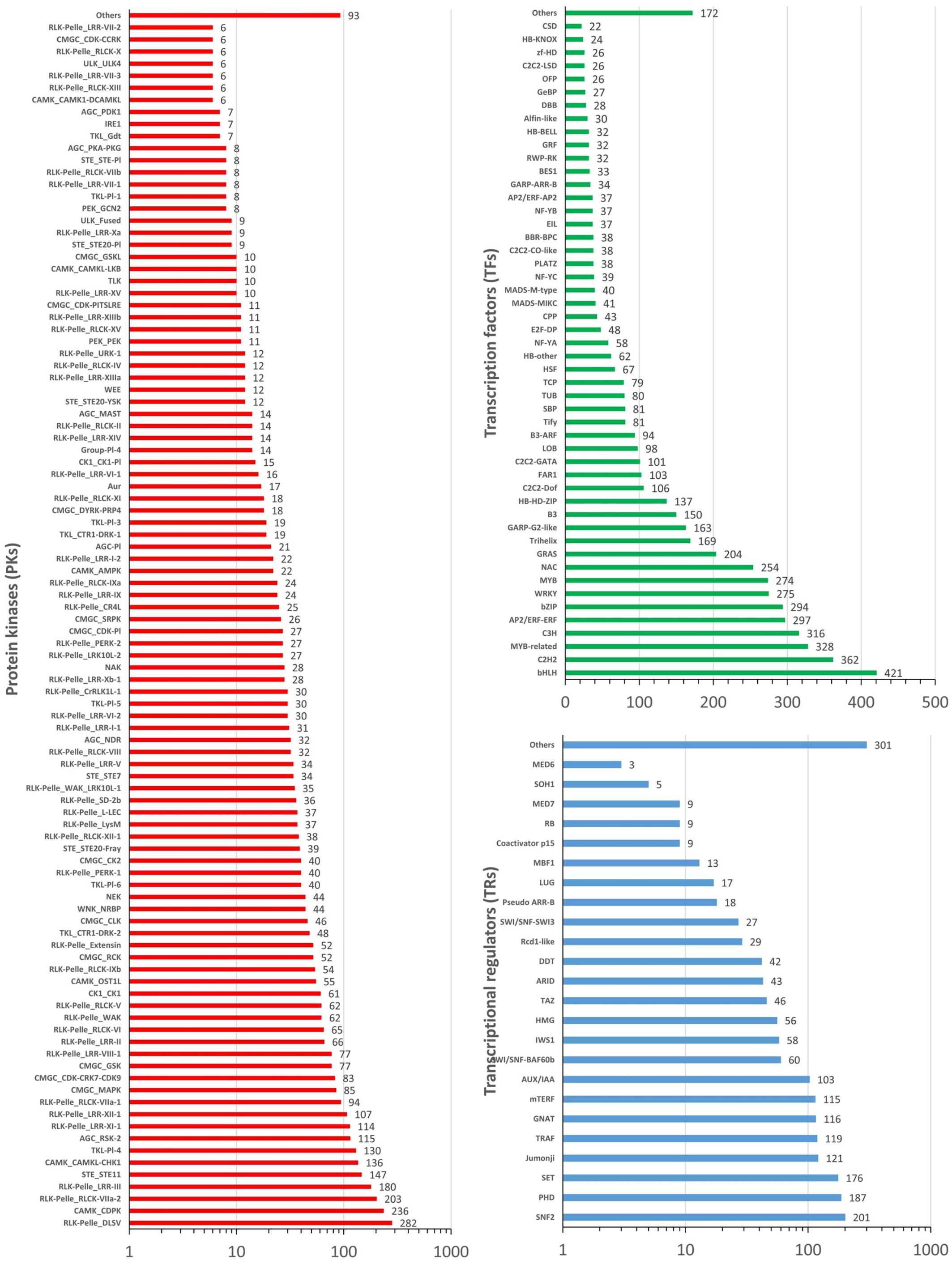

Supplementary Figure 4. Protein kinases (PKs), transcription factors (TFs), and transcription regulators (TRs) in the *D. lanata* transcriptome.

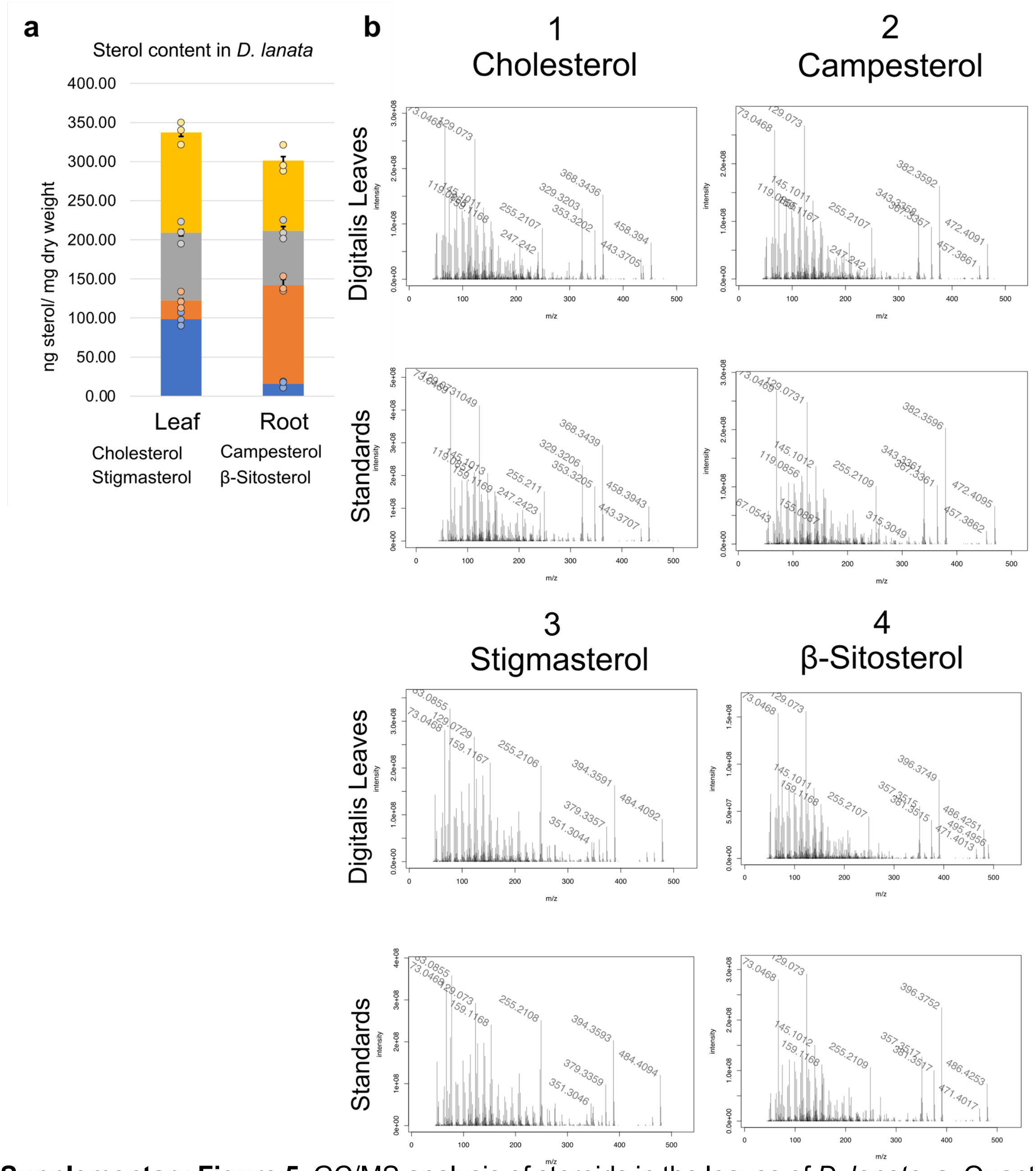

Supplementary Figure 5. GC/MS analysis of steroids in the leaves of *D. lanata*. **a.** Quantification of sterol contents in *D. lanata* leaves and roots. The sterols are quantified according to Itkin, M. et. al.[23] Data represent the average ± SD of three biological replicates. **b.** MS spectra of sterols present in *D. lanata* leaves (top) compared to spectra of standards (bottom). All detected m/z values of the samples are within 5 ppm that of the standards. Compound 1: cholesterol-TMS 458.8347 $[M]^+$; compound 2: campesterol-TMS 472.8612 $[M]^+$; compound 3: stigmasterol-TMS 484.8719 $[M]^+$; compound 4: *β*-sitosterol-TMS 486.8878 $[M]^+$.

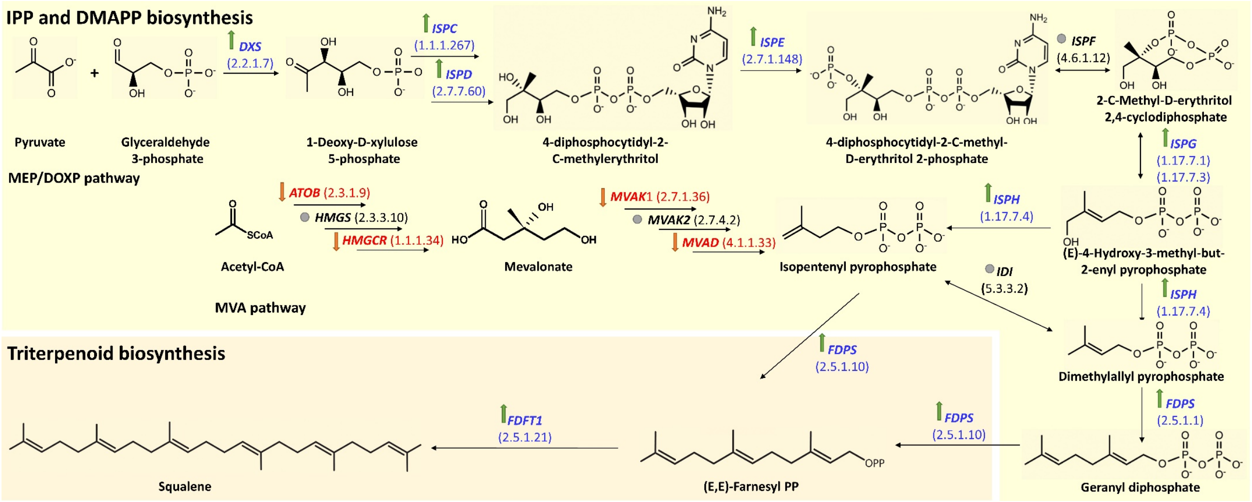

Supplementary Figure 6. Differential expression of terpenoid biosynthetic pathways in leaves vs. roots. The green upward arrow denotes higher expression in leaf versus root; the orange downward arrow conversely denotes higher expression in roots versus leaves; the gray circle denotes almost the same level of expression in roots and leaves. *DXS*: 1-deoxy-D-xylulose-5-phosphate synthase, *ISPC*: 1-deoxy-D-xylulose-5-phosphate reductoisomerase, *ISPD*: 2-C-methyl-D-erythritol 4-phosphate cytidylyltransferase, *ISPE*: 4-diphosphocytidyl-2-C-methyl-D-erythritol kinase, *ISPF*: 2-C-methyl-D-erythritol 2,4-cyclodiphosphate synthase, *ISPG*: (E)-4-hydroxy-3-methylbut-2-enyl-diphosphate synthase, *ISPH*: 4-hydroxy-3-methylbut-2-en-1-yl diphosphate reductase, *HMGS*: hydroxymethylglutaryl-CoA synthase, *MVAK2*: phosphomevalonate kinase, *IDI*: isopentenyl-diphosphate delta-isomerase, *FDPS*: farnesyl diphosphate synthase, *FDFT1*: farnesyl-diphosphate farnesyltransferase.

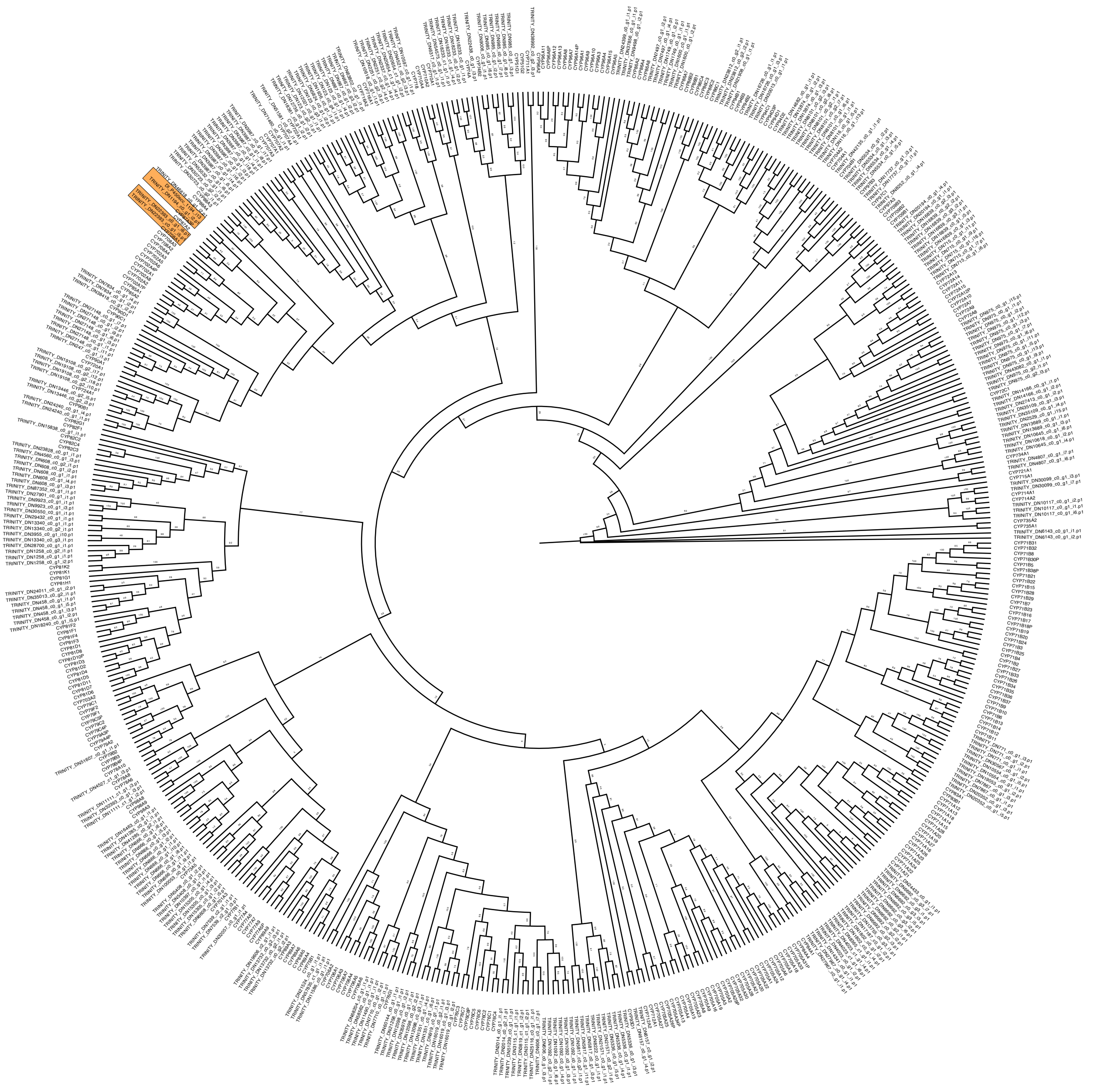

Supplementary Figure 7. Phylogenetic tree with *D. lanata* and *Arabidopsis thaliana* cytochrome P450s. *D. lanata* sequences include transcripts that encode proteins containing the Pfam domain PF00067-CYPs and between 400-600 amino acids long. The *Arabidopsis* proteins were taken from the *Arabidopsis* cytochrome P450 database [24]. The tree was made with RaxML-NG with proteins aligned in MAFFT.

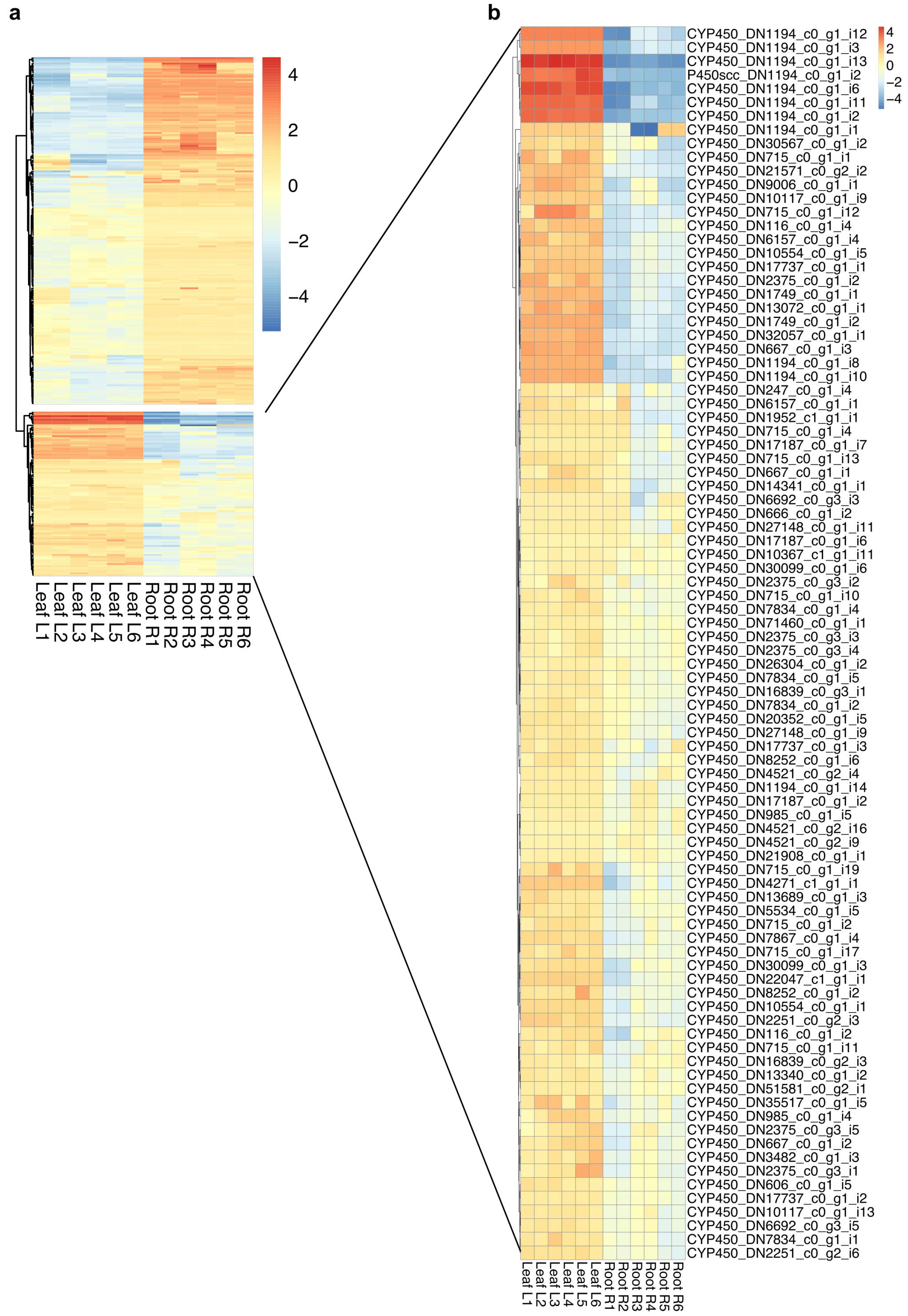

Supplementary Figure 8. Differentially expressed cytochrome P450s in the leaves and roots of *D. lanata*. **a.** Heatmap of 294 transcripts of cytochrome P450s that are differentially expressed in the leaves and root tissues. Results include six biological and technical replicates. **b.** 104 cytochrome P450 transcripts are over-expressed in leaves than in roots.

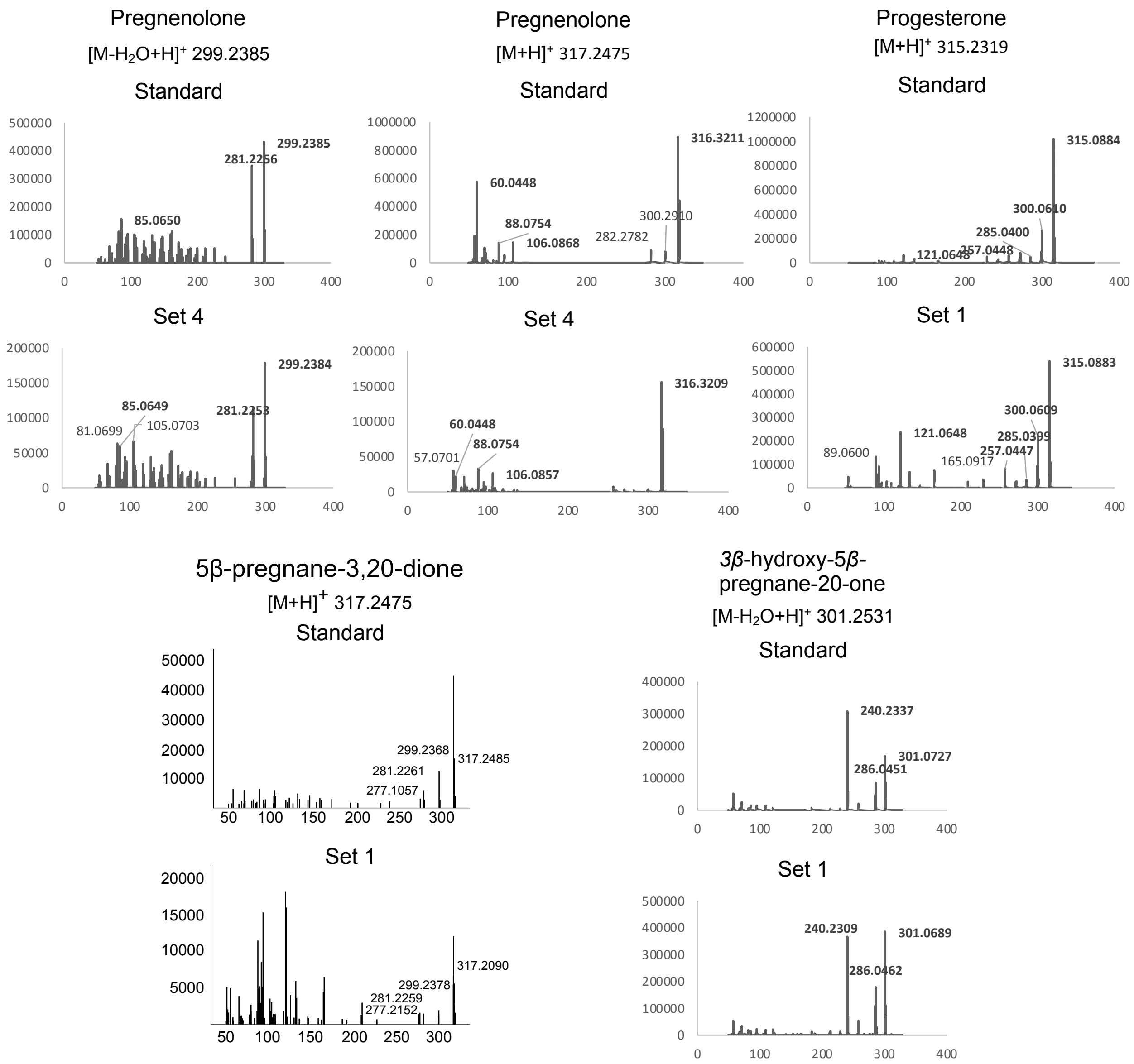

Supplementary Figure 9. MS spectra of pathway intermediates in tobacco transiently expressing digoxin biosynthetic genes in Figure 2C. The theoretical m/z values of parent ion adducts are given. All detected m/z values of the samples are within 5 ppm that of the theoretical values.

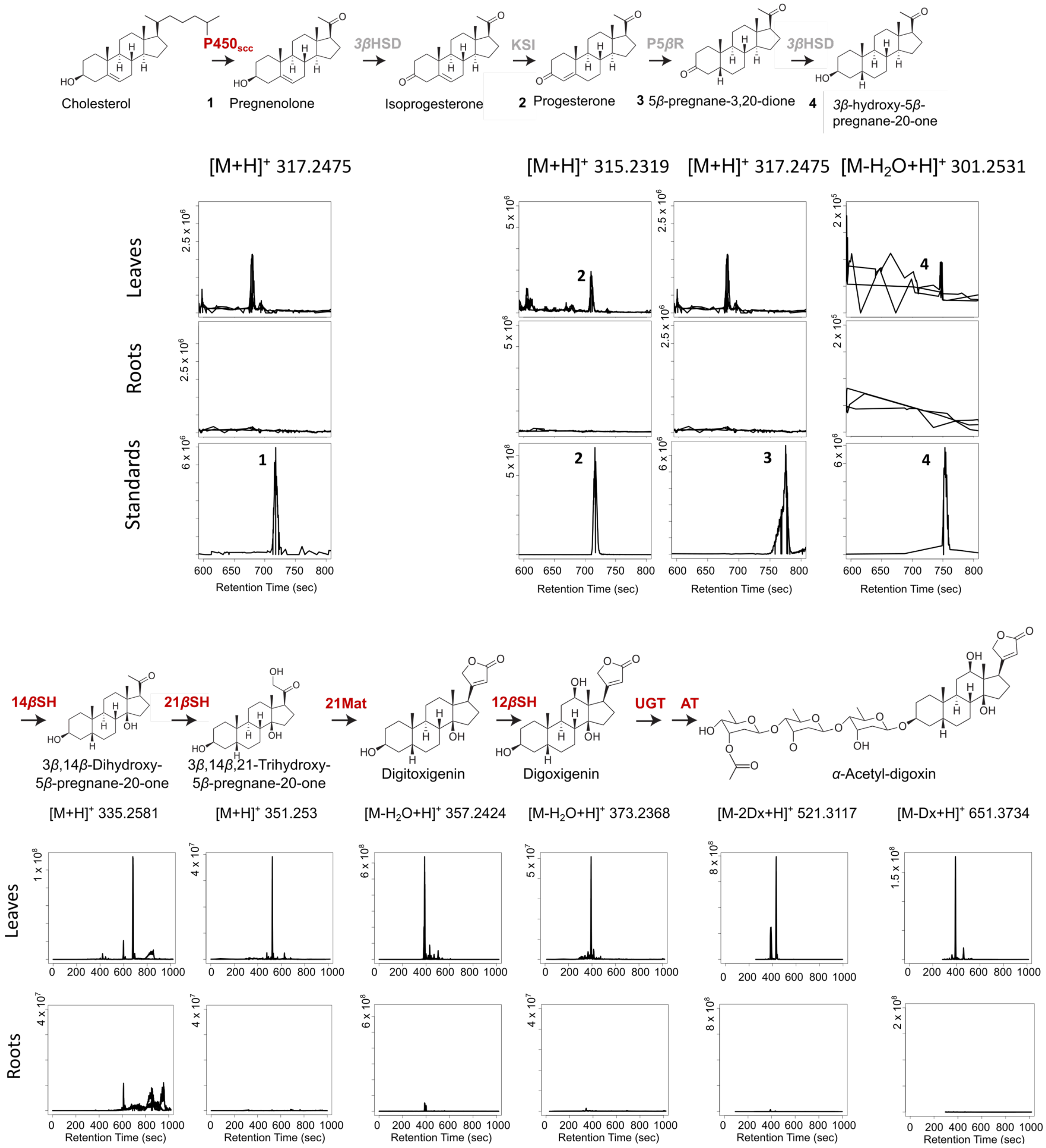

Supplementary Figure 10. Identifying putative digoxin pathway intermediates in *D. lanata*. Extracted ion chromatograms for each compound in the pathway. The theoretical m/z values of parent ion adducts are given. All detected m/z values of the samples are within 5 ppm that of the theoretical values. Standards are provided for pregnenolone (1), progesterone (2), 5*β*-pregnane-3,20-one (3), and 3*β*-hydroxy-5*β*-pregnane-20-one (4). Dx: digitoxose unit.

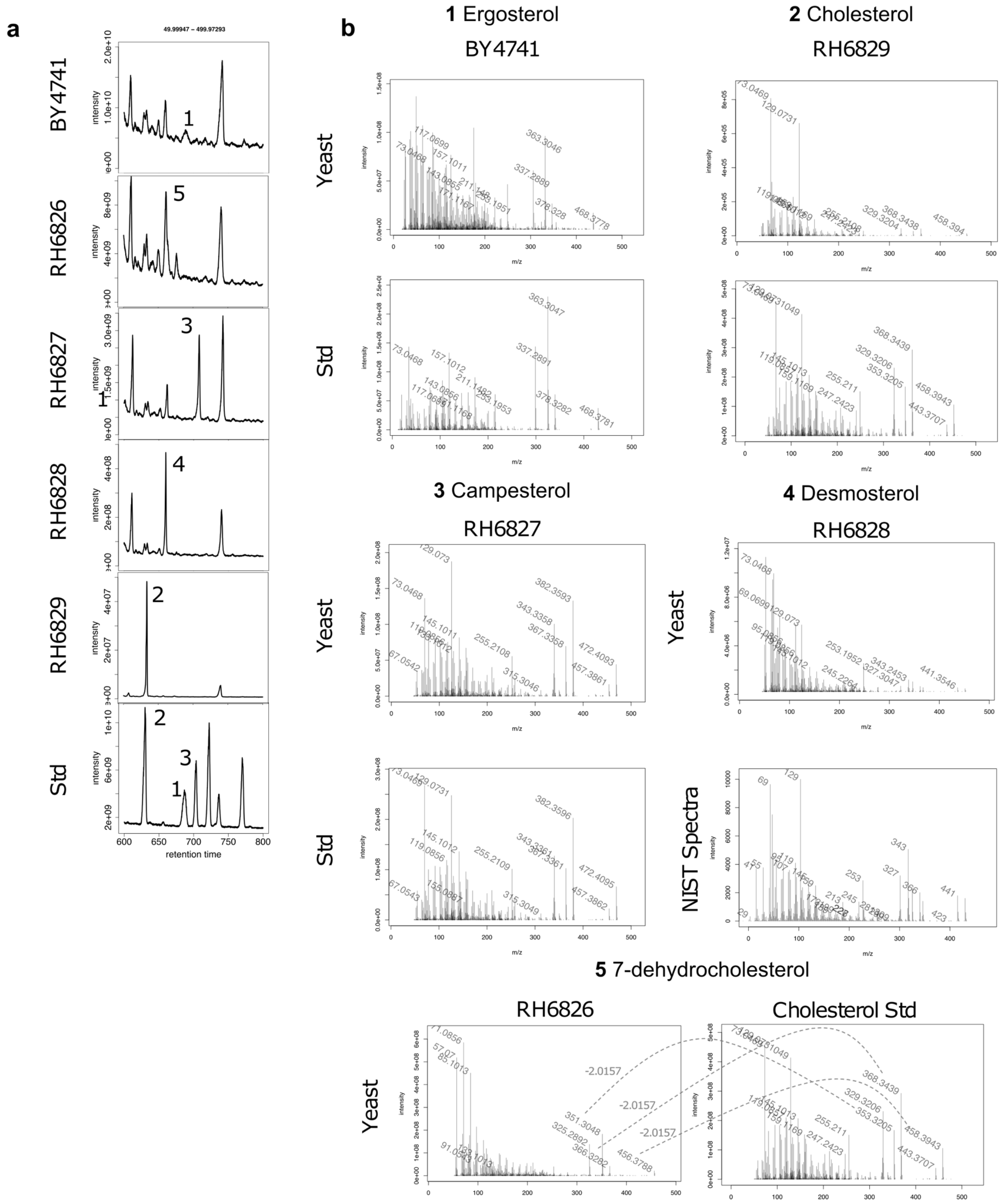

Supplementary Figure 11. GC/MS analysis of sterol contents in engineered yeast strains. BY4741: wildtype yeast containing ergosterol; RH6826: 7-dehydrocholesterol producing yeast, RH6827: campesterol-producing yeast; RH6828: desmosterol-producing yeast; RH6829: cholesterol-producing yeast. **a.** Total ion chromatogram of yeast strains compared to authentic standards. Compound 1: ergosterol-TMS 468.8295 $[M]^+$; compound 2: cholesterol-TMS 458.8347 $[M]^+$; compound 3: campesterol-TMS 472.8612 $[M]^+$; compound 4: desmosterol-TMS 456.8188 $[M]^+$; compound 5: 7-dehydrocholesterol-TMS 442.7922 $[M]^+$. **b.** MS spectra of indicated peaks compared to authentic standards or spectra in the NIST database. All detected m/z values of the samples are within 5 ppm that of the theoretical values.

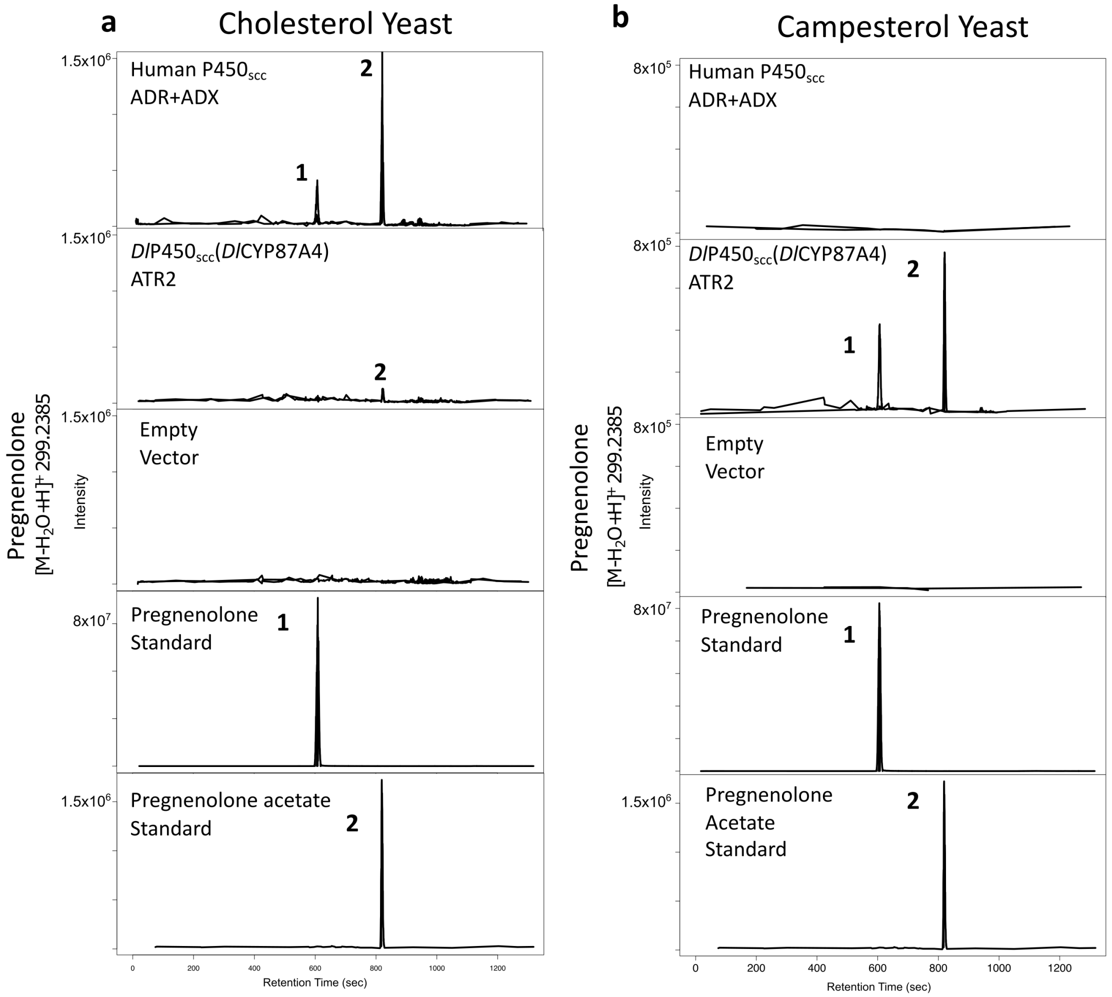

Supplementary Figure 12. Preliminary experiment with campesterol-producing yeast expressing *Dl*CYP87A4. **a.** Cholesterol-producing yeast strain (RH6829) expressing either the human $P450_{scc}$ or the *Digitalis* $P450_{scc}$ with their respective redox partners. Yeast was harvested after growing in a shake flask for 21 hours, followed by extraction and LC/MS. ADX: adrenodoxin, ADR: adrenodoxin reductase. ATR2: *Arabidopsis* cytochrome P450 reductase 2. **b.** Campesterol-producing yeast strain (RH6827) expressing either the human $P450_{scc}$ or the *Digitalis* $P450_{scc}$ with their respective redox partners. Yeast was harvested after growing in a shake flask for 18 hours, followed by extraction and LC/MS. Compound 1: pregnenolone; compound 2: pregnenolone acetate. . The theoretical m/z values of parent ion adducts are given. All detected monoisotopic m/z values of the samples are within 5 ppm that of the theoretical values.

```
                                1
Homo_sapiens_CYP11A1            MLAKGLPPRS VLVKGCQTFL SAPREGLGRL RVPTGEGAGI STRSP--RPF
Bos_taurus_CYP11A1              ---------- ---------- ---------- --------MA STKTP--RPY
Digitalis_lanata_CYP87A4        ---------- -------MSL VAMSVGAILI IITIITNLVF KWWNRSLSSG
Digitalis_lanata_CYP87A3        ---------- -------MSL VAMSVGAILI IITIITNLVF KWWNRSLSSG
Digitalis_purpurea_CYP87A       ---------- ---------- ---------- ---------- ----------
Digitalis_lanata_CYP87A1        ---------- -------MIA AAMYIVAISS LVMIISNWVY RWWNP--SCN
Digitalis_lanata_CYP87A2        ---------- -------MFS VVMYIAAI-- LIVIISNWLY KWWNP--SCN
Arabidopsis_thaliana_CYP87A2    ---------- -------MWA LLIWVSLL-- -LISITHWVY SWRNP--KCR
Ipomoea_indica_CYP87A           ---------- ---------- --MCIGAL-- IFVIITHWIY RWSNP--RCK
Pinguicula_agnata_CYP87A        ---------- -------MVA IAMCIVTLLV TLVTL-NWLY KWYNP--KCN

                               51
Homo_sapiens_CYP11A1            NEIPSPGDNG WLNL---YHF WRETGTHKVH LHHVQNFQKY GPIYREKLGN
Bos_taurus_CYP11A1              SEIPSPGDNG WLNL---YHF WREKGSQRIH FRHIENFQKY GPIYREKLGN
Digitalis_lanata_CYP87A4        GVLP-PGSFG WPLIGETLHF FTPNTSFDVT PFVKDRMKRY GPIFKTSLVG
Digitalis_lanata_CYP87A3        GVLP-PGSFG WPLIGETLHF FTPNTSFDVT PFVKDRMKRY GPIFKTSLVG
Digitalis_purpurea_CYP87A       ---------- ---------- ---------- ------MKRY GPIFKTSLVG
Digitalis_lanata_CYP87A1        GVLP-PGSMG WPLIGETLCF FAPNTSCDVS PFVKERMKRY GSIFKTSLVG
Digitalis_lanata_CYP87A2        GVLP-PGSMG WPLIGETLCF FAPNTSCDVS PFVKERMKRY GSIFKTSLVG
Arabidopsis_thaliana_CYP87A2    GKLP-PGSMG FPLLGESIQF FKPNKTSDIP PFIKERVKKY GPIFKTNLVG
Ipomoea_indica_CYP87A           GKLP-PGSMG WPLLGETLQF FAPNSTSDIP PFVKERMKRY GPIFRTSLVG
Pinguicula_agnata_CYP87A        GTLP-PGSMG WPLLGETLQF FAPNKTFDIQ PFVKKRMQRY GPIFKTSIVR

                              101
Homo_sapiens_CYP11A1            VESVYVIDPE DVALLFKSEG P-----NPER FLIPPWVAYH QYYQRPIGVL
Bos_taurus_CYP11A1              LESVYIIHPE DVAHLFKFEG S-----YPER YDIPPWLAYH RYYQKPIGVL
Digitalis_lanata_CYP87A4        VPVIVSTDAE LNNFIFQQEG QTFQSWYPST FT-------E IFGRENLSTL
Digitalis_lanata_CYP87A3        VPVIVSTDAD LNNFIFQQEG QTFQSWYPST FT-------E IFGRENLSTL
Digitalis_purpurea_CYP87A       VPVIVSTDAE LNNFIFQQEG QTFQSWYPST FT-------E IFGRENLSTL
Digitalis_lanata_CYP87A1        RPVIVSTDAD LNYYIFQQEG QLFQSWYPDT FT-------E IFGRQNVGSL
Digitalis_lanata_CYP87A2        RPVIVSTDAD LNYYIFQQEG QLFQSWYPDT FT-------E IFGRQNVGSL
Arabidopsis_thaliana_CYP87A2    RPVIVSTDAD LSYFVFNQEG RCFQSWYPDT FT-------H IFGKKNVGSL
Ipomoea_indica_CYP87A           RPVIVSTDPE LNYFIFQQEG QLFQSWYPDT FT-------E IFGRQNVGSL
Pinguicula_agnata_CYP87A        QPLIVSTDAE FNHFIFQQEG QLFQSWYPDT FT-------E VFGKENLGTL

                              151
Homo_sapiens_CYP11A1            LKKSAAWKKD RVALNQEVMA PEATKNFLPL LDAVSRDFVS VLHRRIKKAG
Bos_taurus_CYP11A1              FKKSGTWKKD RVVLNTEVMA PEAIKNFIPL LNPVSQDFVS LLHKRIKQQG
Digitalis_lanata_CYP87A4        HGFMYKYFKN MVL---GLFG PESLKTMISE VENTS----- --NINLKRWS
Digitalis_lanata_CYP87A3        HGFMYKYFKN MVL---GLFG PESLKTMISE VETTS----- --NINLKRW-
Digitalis_purpurea_CYP87A       HGFMYKYFKN MVL---GLFG PESLKTMISE VENTS----- --NINLKRW-
Digitalis_lanata_CYP87A1        HGFMYKYLKN TVL---NLFG PESLKKMIPE FEHAS----- --NRSLMRW-
Digitalis_lanata_CYP87A2        HGFMYKYLKN TVL---NLFG PESLKKMIPE FEQAS----- --NRSLMRW-
Arabidopsis_thaliana_CYP87A2    HGFMYKYLKN MVL---TLFG HDGLKKMLPQ VEMTA----- --NKRLELW-
Ipomoea_indica_CYP87A           HGFMYKYLKN MVL---NLFG PESLKKMLPE VEEAA----- --NRNLRRW-
Pinguicula_agnata_CYP87A        HGFMYKYLKN MVL---NLFG VENLKRMIGE IEHAS----- --AESLKRW-

                              201
Homo_sapiens_CYP11A1            SGNYSGDISD DLFRFAFESI TNVIFGERQG MLEEVVNPEA QRFIDAIYQM
Bos_taurus_CYP11A1              SGKFVGDIKE DLFHFAFESI TNVMFGERLG MLEETVNPEA QKFIDAVYKM
Digitalis_lanata_CYP87A4        SSNGTVELKD AIAEMIFELT AKKLISYELE KSPYNLRDNF VAFIDGL---
Digitalis_lanata_CYP87A3        SENGTIELKD AIAEMIFELT AKKLISYELE KSPYNLRDNF VAFIDGL---
Digitalis_purpurea_CYP87A       SANGTVELKD AIAEMIFELT AKKLISYELE KSPYNLRDNF VAFIDGL---
Digitalis_lanata_CYP87A1        STQSNVEIKN ATAKMIFDLT ARKLISCDSE TSSDDLRKNF VAFIQGL---
Digitalis_lanata_CYP87A2        STQSNVEIKN ATAKMIFDLT ARKLISCDSE TSSDDLRKNF VAFIQGL---
Arabidopsis_thaliana_CYP87A2    SNQDSVELKD ATASMIFDLT AKKLISHDPD KSSENLRANF VAFIQGL---
Ipomoea_indica_CYP87A           SNQTNVEMKE STATMIFDLT AKKLISYDSE NSSENLRESF VAFIQGL---
Pinguicula_agnata_CYP87A        SGLPSVEMKD ATAQMVFDLS AKKLISYDSQ NASADLRDCF ESFIKGL---

                              251
Homo_sapiens_CYP11A1            FHTSVPMLNL PPDLFRLFRT KTWKDHVAAW DVIFSKADIY TQNFYWELRQ
Bos_taurus_CYP11A1              FHTSVPLLNV PPELYRLFRT KTWRDHVAAW DTIFNKAEKY TEIFYQDLRR
Digitalis_lanata_CYP87A4        --ISFP-LNI PGTAY----Y RCLQGRKNAI KMLKDMLHER RE----KPRE
Digitalis_lanata_CYP87A3        --ISFP-LNI PGTAY----Y KCLQGRNNAI KMLKAMLHER RE----KPRE
Digitalis_purpurea_CYP87A       --ISFP-LNI PGTAY----Y KCLQGRKNAI KMLRDMLHER RE----KPRE
Digitalis_lanata_CYP87A1        --ISFP-LNI PGTAY----S RCLKGRKNVM KMLRNMLQER RE----RPQK
Digitalis_lanata_CYP87A2        --ISFP-LNI PGTAY----S RCLKGRKNVM MMLQNMLRER RE----RPQK
Arabidopsis_thaliana_CYP87A2    --ISFP-FDI PGTAY----H KCLQGRAKAM KMLRNMLQER RE----NPRK
Ipomoea_indica_CYP87A           --ISFP-LDI PGTAY----H NCLQGRKKAM KLLKEKIEER RA----NPRK
Pinguicula_agnata_CYP87A        --ISFP-LNI PGTAY----N RCMQGRNKAM TMLKSMLQER RE----RPRK
```

```
                                  301
Homo_sapiens_CYP11A1              KGSVHHDYRG ILYRLLGDSK MSFEDIKANV T-EMLAGGVD TTSMTLQWHL
Bos_taurus_CYP11A1                K-TEFRNYPG ILYCLLKSEK MLLEDVKANI T-EMLAGGVN TTSMTLQWHL
Digitalis_lanata_CYP87A4          TQTDFFDY-- VLEELQKEDT IITETLALDL MFVLLFASHE TASIALTLAM
Digitalis_lanata_CYP87A3          TQTDFFDY-- VLEEFQKEDT IITETLALDL MFVLLFASHE TASIALTLAM
Digitalis_purpurea_CYP87A         TQTDFFDY-- VLEELQKQDT IITETLALDL MFVLLFASHE TASIALTLAM
Digitalis_lanata_CYP87A1          SQADFFDY-- VLEELQREDT PLTEAIALDL MFVLLFASYE TTSLALTLAI
Digitalis_lanata_CYP87A2          NQADFFDY-- VLAELQREDT PLTEAIALDL MFVLLFASYE TTSLALTLAI
Arabidopsis_thaliana_CYP87A2      NPSDFFDY-- VIEEIQKEGT ILTEEIALDL MFVLLFASFE TTSLALTLAI
Ipomoea_indica_CYP87A             NQTDFFDF-- VLEELKRKDT ILTEGIALDL MFVLLFASFE TTSLALTLAL
Pinguicula_agnata_CYP87A          EQSDFFDY-- VLVELQREDT LMTESIALDL MFVLLFASYE TTSLALTLGM

                                  351
Homo_sapiens_CYP11A1              YEMARNLKVQ DMLRAE---V LAARHQAQGD MA-TMLQLVP LLKASIKETL
Bos_taurus_CYP11A1                YEMARSLNVQ EMLREE---V LNARRQAEGD IS-KMLQMVP LLKASIKETL
Digitalis_lanata_CYP87A4          KFLVDHPLVL EKLTEEHDEI IKTREDPNSG LTWNEYKSMK FTFQFINETL
Digitalis_lanata_CYP87A3          KFLVDHPLVL EKLTEEHDEI IKTREDPNSG LTWNEYKSMK FTFQFINETL
Digitalis_purpurea_CYP87A         KFLVDHPLVL DKLTEEHDEI IKMREDPNSG LTWNEYKSMK FTFQFINETL
Digitalis_lanata_CYP87A1          KFLLERPSVL KELTEEHEAI IRRRENPDSE LTWTEYKSMR FTFQVINETV
Digitalis_lanata_CYP87A2          KFLLEHPSVL EKLAEEHEAI IRRRENPDSG LTWTEYKSMR FTFQFINETA
Arabidopsis_thaliana_CYP87A2      KFLSDDPEVL KRLTEEHETI LRNREDADSG LTWEEYKSMT YTFQFINETA
Ipomoea_indica_CYP87A             KFLSDHPLVL EKLTEEHEAI IKNRENPNSG LTWQEYKSMT FTFQFIHETV
Pinguicula_agnata_CYP87A          RFLVENEQVL RELQHEHEAI IKQREHPDSG LTWAEYKSMK FTFQFINETV

                                  401
Homo_sapiens_CYP11A1              RLHPISVTLQ RYLVNDLVLR DYMIPAKTLV QVAIYALGRE PTFFFDPENF
Bos_taurus_CYP11A1                RLHPISVTLQ RYPESDLVLQ DYLIPAKTLV QVAIYAMGRD PAFFSSPDKF
Digitalis_lanata_CYP87A4          RLANIAPLIF RKALTETEFK GYTIPAGWAV MVCLPAVHLD PSKYKNPLEF
Digitalis_lanata_CYP87A3          RLANIAPLIF RKALTETEFK GFTIPAGWAV MVCLPAVHLD PTKYKNPLEF
Digitalis_purpurea_CYP87A         RLANIAPLIF RKALTETEFK GYTIPAGWAV MVCLPAVHLD PTKYKNPLEF
Digitalis_lanata_CYP87A1          RLANIVPAIF RKALRETVFK GYTIPAGWAV MVCPPAVHLN PAKYKDPLEF
Digitalis_lanata_CYP87A2          RLANIVPAIF RKALRETVFK GYTIPAGWAV MVCPPAVHLN PEKYKDPLEF
Arabidopsis_thaliana_CYP87A2      RLANIVPAIF RKALRDIKFK DYTIPAGWAV MVCPPAVHLN PEMYKDPLVF
Ipomoea_indica_CYP87A             RLANIVPGIF RKSLRDINFK GYTIPAGWAV MVCPPAVHLN PTRYKDPLEF
Pinguicula_agnata_CYP87A          RLANIAPGIF RKTMTDIQYK EYTIPAGWAV VACPPAVHLN PDKYVNPLEF

                                  451
Homo_sapiens_CYP11A1              DPTRWLSKDK NITYFRNLGF GWGVRQCLGR RIAELEMTIF LINMLENFRV
Bos_taurus_CYP11A1                DPTRWLSKDK DLIHFRNLGF GWGVRQCVGR RIAELEMTLF LIHILENFKV
Digitalis_lanata_CYP87A4          NPWRWEGVDT SVGSKTFMAF GGGMRLCIGA DFTKVQMAVF LHCLVTKYKW
Digitalis_lanata_CYP87A3          NPWRWEGVDT SVGSKTFMAF GGGMRLCIGA DFTKVQMAVF LHCLVTKYKW
Digitalis_purpurea_CYP87A         NPWRWEGVDT SVGSKTFMAF GGGMRLCIGA DFTKVQMAVF LHCLVTKYKW
Digitalis_lanata_CYP87A1          NPWRWEGVDT NGASRNFMAF GGGMRFCVGT EFTKVQMAVF LHCLVTKYKF
Digitalis_lanata_CYP87A2          NPWRWEGVDT NGASRNFMAF GGGMRFCVGT EFTKVQMAVF LHCLVTKYKL
Arabidopsis_thaliana_CYP87A2      NPSRWEGSKV TNASKHFMAF GGGMRFCVGT DFTKLQMAAF LHSLVTKYRW
Ipomoea_indica_CYP87A             NPWRWEGVEI NGATRNFMAF GGGMRFCVGT DFTKVQMAVF LHCLVTKYKW
Pinguicula_agnata_CYP87A          NPGRWEGIDT NGGSKHFMAF GGGMRFCVGA EFSKLQMAVF VHCLVTKYKF

                                  501
Homo_sapiens_CYP11A1              EIQHLSDVGT TFNLILMPEK PISFTFWPFN QEATQQ----
Bos_taurus_CYP11A1                EMQHIGDVDT IFNLILTPDK PIFLVFRPFN QDPPQAHHHH
Digitalis_lanata_CYP87A4          KSIKGGDIVR CPGLKF---- PNGFHVNMTE RG*-------
Digitalis_lanata_CYP87A3          KSIKGGDIVR SPGLKF---- PNGFH----- ----------
Digitalis_purpurea_CYP87A         KTVKGGDIVR CPGLKF---- PNGFHVHMTE REVNKMACK-
Digitalis_lanata_CYP87A1          KAIKGGDIVR TPGLQF---- PNGFHIQISE KER*------
Digitalis_lanata_CYP87A2          KAIKGGDIVR TPGLQF---- PNGFHIQISE KDG*------
Arabidopsis_thaliana_CYP87A2      EEIKGGNITR TPGLQF---- PNGYHVKLHK KRD-------
Ipomoea_indica_CYP87A             IPIKGGDILR TPGLQF---- PNGFHIQISE K---------
Pinguicula_agnata_CYP87A          KAIKGGETIR TPGLQF---- PNGYHIQMSG K---------
```

Supplementary Figure 13. Multi-sequence alignment of *Dl*CYP87A4 with mammalian CYP11A1 and other plant CYP87As.

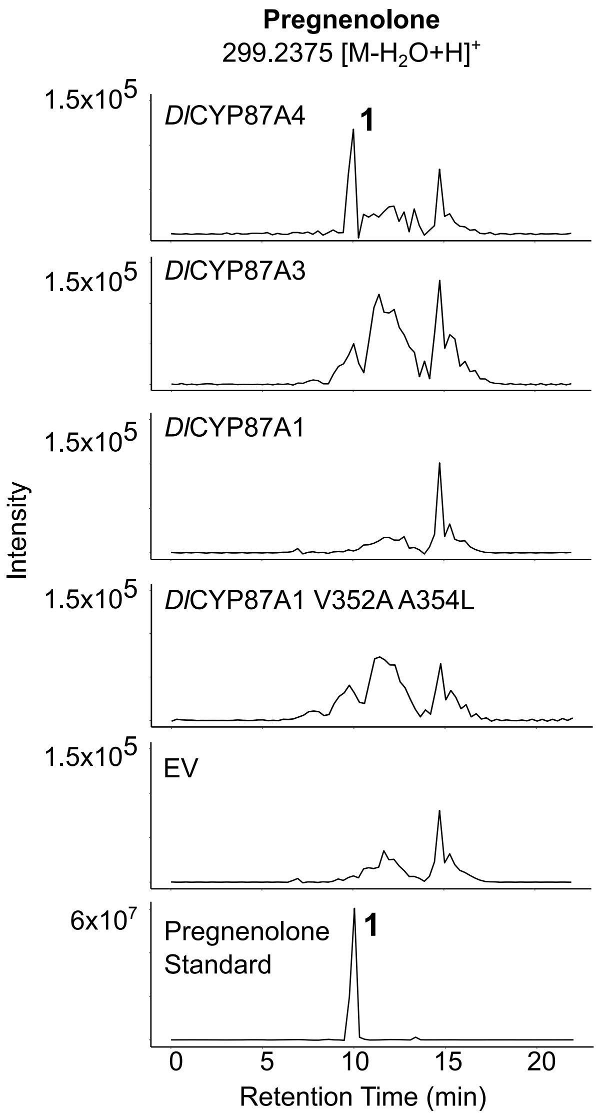

Supplementary Figure 14: Extracted ion chromatogram for pregnenolone from the campesterol-producing yeast strain expressing various *Digitalis lanata* CYP87As and the ATR2 redox partner. The theoretical m/z value of the pregnenolone adduct is given. All detected m/z values of the samples are within 5 ppm that of the theoretical values.

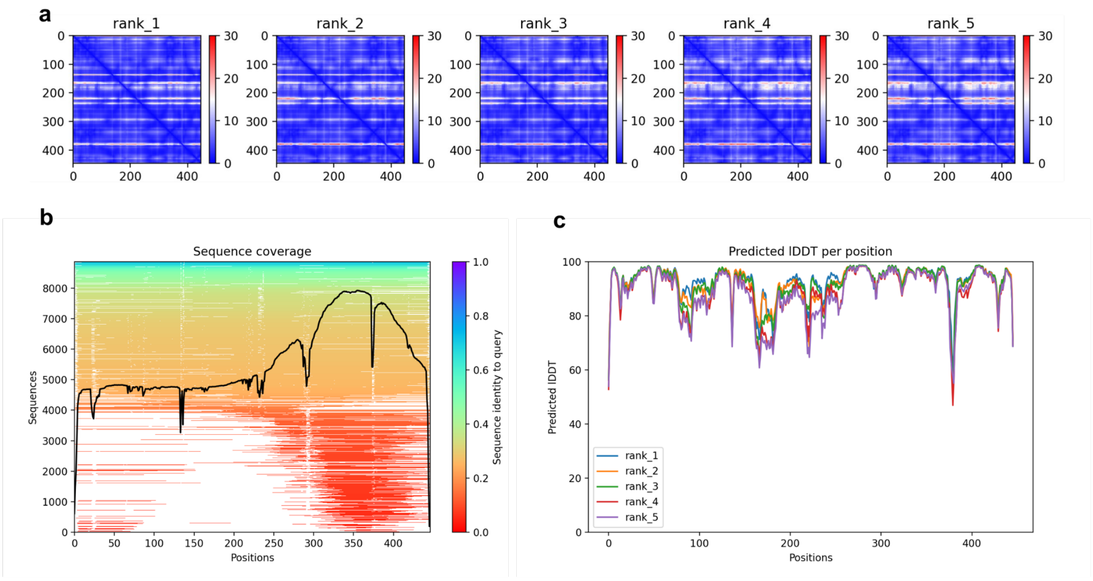

Supplementary Figure 15. Metadata from Alphafold2 modeling of *Dl*CYP87A4. The protein sequence is truncated of the N-terminal signal peptide with 31 amino acids. Five models were generated. The model with the highest ranking predicted aligned error (PAE) was chosen for docking. PAE (**a**), sequence coverage (**b**), and predicted local distance difference test (pLDDT) (**c**) per position for each of the five models. pLDDT >70 is confidently predicted amino acid positions.

Supplementary Tables

Supplementary Table 1. Top 20 simple sequence repeats (SSRs) in a total of 22,549 *D. lanata* SSRs. More than 92% of the total non-redundant SSRs are represented in the above table.

SSR Motif	No. of repeats												
	4	5	6	7	8	9	10	11	12	13	14	15	>15	Total
**AG/CT**	-	-	1503	956	675	453	277	212	142	84	92	73	434	4901
**AT/AT**	-	-	1268	748	626	525	318	527	665	-	-	-	-	4677
**AC/GT**	-	-	1093	603	393	293	186	143	99	26	25	20	54	2935
**AAG/CTT**	-	776	382	185	80	39	30	28	16	36	22	11	42	1647
**AAT/ATT**	-	704	404	171	150	11	35	20	25	13	26	14	27	1600
**ATC/ATG**	-	564	225	129	78	28	11	3	2	4	5	5	13	1067
**ACC/GGT**	-	505	178	42	23	1	2	-	-	-	-	-	2	753
**AGC/CTG**	-	510	193	17	17	1	-	-	-	-	-	-	-	738
**AGG/CCT**	-	366	126	71	28	4	7	9	6	1	-	-	-	618
**AAC/GTT**	-	179	98	42	25	4	5	5	7	-	-	2	5	372
**ACT/AGT**	-	164	83	27	19	15	1	5	-	-	-	-	-	314
**CCG/CGG**	-	222	37	19	-	-	-	-	-	1	-	-	-	279
**ACAT/ATGT**	-	88	39	9	8	16	21	1	3	11	-	-	-	196
**AAAT/ATTT**	-	109	33	2	2	3	4	1	-	-	-	-	-	154
**AAAAT/ATTTT**	99	18	1	-	1	2	-	-	-	-	-	-	-	121
**AAACC/GGTTT**	82	29	1	4	1	1	-	-	-	-	-	-	-	118
**ACG/CGT**	-	82	14	6	-	-	-	-	-	-	-	-	-	102
**AAAAG/CTTTT**	63	10	-	1	-	-	-	5	-	-	-	-	-	79
**AAAG/CTTT**	-	36	12	5	-	-	2	-	-	-	-	-	-	55
**AAAAC/GTTTT**	36	9	2	-	-	-	-	-	1	-	-	-	-	48
													**Total**	20774

Supplementary Table 2. *Dl*CYP87A4 with closely related cytochrome P450s in various plant species. Aa: amino acid.

Query	Subject	Species	% Identical (aa)	Alignment Length	Mismatches	Gap Opens	E Value	Bit Score
*Dl*CYP87A4	dpa_locus_4690_iso_4_len_1619_ver_1	*Digitalis purpurea*	97.30	408	10	1	0	827
*Dl*CYP87A4	XP_011081025.1	*Sesamum indicum*	74.42	473	116	3	0	747
DlCYP87A4	XP_022881263.1	*Olea europaea*	74.63	469	114	3	0	744
*Dl*CYP87A4	*Dl*CYP87A1	*Digitalis lanata*	72.27	476	129	2	0	727
*Dl*CYP87A4	cal_g005561.t1	*Calotropis gigantea*	68.99	445	133	3	0	636
*Dl*CYP87A4	m_44857	*Asclepias syriaca*	52.49	482	214	6	0	530
*Dl*CYP87A4	m_12618	*Asclepias syriaca*	38.57	446	263	7	2.38E-111	330
*Dl*CYP87A4	*Dl*CYP87A3	*Digitalis lanata*	97.45	470	11	1	0	891
*Dl*CYP87A4	*Dl*CYP87A2	*Digitalis lanata*	74.18	457	115	2	0	692

Supplementary Table 3. Primers used in this study. FWD/FW/F: forward primers, REV/RW/R: reverse primers. Capital letters: gene-specific nucleotides; Small case letters: additional nucleotides for cloning.

**Name**	**Sequence**
**Primers for cloning genes into the pEAQ vector by Gibson Cloning**	
TRINITY_DN247_c0_g1_i1.p1 FWD	tattctgcccaaattcgcgaATGGACTCCAACATGTTTCTCTAC
TRINITY_DN247_c0_g1_i1.p1 REV	accagagttaaaggcctcgaTCACTTAAGCCTCTGAATGATAATCG
TRINITY_DN1194_c0_g1_i13.p1 FWD	tattctgcccaaattcgcgaATGTCGTTAGTAGCTATGAGC
TRINITY_DN1194_c0_g1_i13.p1 REV	accagagttaaaggcctcgaTTAGCCTCTCTCAGTCATGT
3βHSD_FWD	tattctgcccaaattcgcgaATGTCGTCAAAGCCAAGGTTGG
3βHSD_REV	accagagttaaaggcctcgaCTAACGCACGACGGTGAAGC
P5βR-2_FWD	tattctgcccaaattcgcgaATGTATACCGACACAACGACT
P5βR-2_REV	accagagttaaaggcctcgaTCAAGGGACAAATCTATAAGATCTCA
**Primers for Golden Gate cloning and domestication into the pYTK001 vector**	
1194 + pYTK001 F	tttcgtctcatcggggtctcatATGTCGTTAGTAGCTATGAGC
1194 sub 1 R	TCCCGTCTCTCATGTAGCATA
1194 sub 2 F	tttcgtctctCATGAGAGgCGGGAAAAGC
1194 sub 2 R	tttcgtctcAGTGAGAGCTTTT
1194 sub 3 F	tttcgtctctTCACTGAaACCGAATTTAAAGG
1194 +pYTK001 R	tttcgtctcaggtcggtctcaggaTTAGCCTCTCTCAGTCATGT
**Primers for qRTPCR**	
TRINITY_DN1194_c0_g1_i13.p1 1110-1131 FWD 1	ATACACAATTCCAGCTGGTTGG
TRINITY_DN1194_c0_g1_i13.p1 1285-1306 REV 1	TCTGGACCTTTGTGAAATCTGC
DN247 qRT FW	CCACACGCATTGGCTCATCTTAAG
DN247 qRT RW	CGACACATTGAGTGAACGGCATAG

Supplementary Table 4. Constructs used in this study. The DNA parts for transcription-unit-level and multi-gene-level plasmids are described in Lee et. al (2015) [21].

**Plasmids**	**Description**
pEAQ-HT_*3βHSD*	pEAQ-HT vector cloned with *D. lanata* gene "*3βHSD*". Transcript ID: "TRINITY_DN638_c0_g3_i1.p1"
pEAQ-HT_*P5βR2*	pEAQ-HT vector cloned with *D. lanata* gene " *P5βR2*". NCBI accession: ADL28122.1
pEAQ-HT_*DN247_P450*	pEAQ-HT vector cloned with *D. lanata* gene *CYP90A1*. Transcript ID: "TRINITY_DN247_c0_g1_i1.p1"
pEAQ-HT_*CYP87A4*	pEAQ-HT vector cloned with *D. lanata CYP87A4* $P450_{scc}$.
pEAQ-HT_*GFP*	pEAQ-HT vector cloned with the green fluorescent protein
pYTK001_$HsP450_{scc}$	Human $P450_{scc}$, or *CYP11A1*, codon-optimized for yeast expression cloned into pYTK001
pYTK001_*HsADX*	Human andrenodoxin (*ADX*), codon-optimized for yeast expression cloned into pYTK001
pYTK001_*HsADR*	Human andrenodoxin reductase (*ADR)*, codon-optimized for yeast expression cloned into pYTK001
pYTK001_*CYP87A4*	*Digitalis lanata* $P450_{scc}$ CYP87A4 domesticated for cloning into the pYTK001
pYTK001_*ATR2*	*Arabidopsis thaliana* cytochrome P450 reductase 2 (*ATR2*) cloned into pYTK001
pTU11S1_*CYP87A4*	Transcription-unit-level plasmid of *D. lanata CYP87A4* with the P_{TDH3} promoter, the T_{ENO1} terminator, and LS-R1 connectors
pTU1116_*ATR2*	Transcription-unit-level plasmid of *ATR2* with the P_{CCW12} promoter, the T_{SSA1} terminator, and L1-RE connectors
pTU11S1_$HsP450_{scc}$	Transcription unit with promoter-CDS-terminator for human $P450_{scc}$ and LE-R1 linker
pTU1112_*HsADX*	Transcription unit with promoter-CDS-terminator for human *ADX* and L1-R2 linker
pTU1126_*HsADR*	Transcription unit with promoter-CDS-terminator for human *ADR* and L2-RE linker
pMGR22_*DlCYP87A4.ATR2*	Multigene plasmid with *D. lanata CYP87A4* TU + *ATR2* TU
pMGR22_*HsP450scc.HsADX.ADR*	Multigene plasmid with human $CPY450_{scc}$ *(CYP11A1)* TU and Human redox partners *ADR* and *ADX*
pYTK001_*DlCYP87A1_DoubleMutant_(DN22393_V352A_A354L)*	Canonical *CYP87A1* from *Digitalis lanata* with two mutations in the active center *DlCYP87A1_DoubleMutant_(DN22393_V352A_A354L)*

pTU11S1_*DlCYP87A1_DoubleMutant_(DN22393_V352A_A354L)*	Transcription unit with the P_{TDH3} promoter, the T_{ENO1} terminator of canonical *CYP87A1* from *Digitalis lanata* with two mutations in the reaction center *DlCYP87A1_DoubleMutant_(DN22393_V352A_A354L)*
pMGR22_*DlCYP87A1_DoubleMutant_(DN22393_V352A_A354L).ATR2*	Multigene plasmid of canonical *CYP87A1* from *Digitalis lanata* with two mutations in the reaction center *DlCYP87A1_DoubleMutant_(DN22393_V352A_A354L)* with the redox partner *ATR2* TU
pYTK001_*DlCYP87A3*	Possible paralog of *DlP450*$_{scc}$ from *Digitalis lanata* and matching with the transcript TRINITY_CYP87A3 cloned into pYTK001
pTU11S1_*DlCYP87A3*	Transcription unit of *Digitalis lanata* CYP87A3 with the P_{TDH3} promoter and the T_{ENO1} terminator
pMGR22_*DlCYP87A3.ATR2*	Multigene plasmid with *Digitalis lanata CYP87A3* TU and *ATR2* TU
pYTK001_*DlCYP87A1_DN22393*	*D. lanata* codon-optimized canonical *DlCYP87A1* cloned into pYTK001
pYTK001_*CYP87A4(A355V)*	*D. lanata* A355V mutated *CYP87A4* cloned into pYTK001
pYTK001_*CYP87A4(L357A)*	*D. lanata* L357A mutated CYP87A4 cloned into pYTK001
pYTK001_*CYP87A4(S123A)*	*D. lanata* S123A mutated CYP87A4 cloned into pYTK001
pTU11S1_*DlCYP87A4(A355V)*	*D. lanata* A355V mutated CYP87A4 cloned into LS-R1 TU vector with the P_{TDH3} promoter and the T_{ENO1} terminator
pTU11S1_*DlCYP87A4(L357A)*	*D. lanata* L357A mutated CYP87A4 cloned into LS-R1 TU vector with the P_{TDH3} promoter and the T_{ENO1} terminator
pTU11S1_*DlCYP87A4(S123A)*	*D. lanata* S123A mutated CYP87A4 cloned into LS-R1 TU vector with the P_{TDH3} promoter and the T_{ENO1} terminator
pTU11S1_*DlDlCYP87A1*	*D. lanata* codon-optimized canonical *DlCYP87A*1 cloned into LS-R1 TU vector with the P_{TDH3} promoter and the T_{ENO1} terminator
pMGR22_*DlCYP87A4(A355V).ATR2*	Multigene plasmid *D. lanata* A355V mutated *CYP87A4* TU + *ATR2* TU
pMGR22_*DlCYP87A4(L357A).ATR2*	Multigene plasmid *D. lanata* L357A mutated *CYP87A4* TU + *ATR2* TU
pMGR22_*DlCYP87A4(S123A).ATR2*	Multigene plasmid *D. lanata* S123A mutated *CYP87A4* TU + *ATR2* TU
pMGR22_*DlCYP87A1.ATR2*	Multigene plasmid *D. lanata* codon-optimized transcript canonical *DlCYP87A1* TU + *ATR2* TU
pMGR22_*EV*	Empty vector backbone for the multigene plasmids

Supplementary Table 5. Yeast strains used in this study. The RH strains are from Dr. Howard Riezman's lab [39].

Strain	Genotype
RH6826	MATa *ura3 leu2 his3 trp1 can1 bar1 erg5Δ::HIS5-TDH3-DHCR24 erg6Δ::TRP1*
RH6827	MATa *ura3 leu2 his3 trp1 can1 bar1 erg5Δ::TRP1-TDH3-DHCR7*
RH6828	MATα *ura3 lue2 trp1 ade2 can1 bar1 erg6Δ::TRP1-TDH3-DHCR7*
RH6829	MATa *ura3 leu2 his3 trp1 can1 bar1 erg5Δ::HIS5-TDH3-DHCR24 erg6Δ::TRP1-TDH3-DHCR7*
BY4741	MATa *his3Δ1 leu2Δ0 met15Δ0 ura3Δ0*

Supplementary Methods

De novo assembly of transcriptome

FastQC (v0.11.5) was used to check the next-generation sequencing data [1]. Then, the Trimmomatic tool embedded in the Trinity transcriptome assembler was used to remove Illumina sequencing adaptors, low-quality leading and trailing bases (below quality 5), cutting a sliding window of size 4 bp when their average quality drops below 5 and also filtering out reads below 25 bp. *De novo* assembly of a single transcriptome (leaf and root) from the raw reads was done using the Trinity assembler program (v2.8.4) at the default k-mer value of 25 [2]. Bowtie2 (v2.3.4.3) was used to map raw reads to the assembled transcripts to ascertain completeness [3]. The overall quality of the transcriptome was ascertained using the N50 and ExN50 values calculated using the Trinity pipeline.

Transcriptome annotation and functional classification

Firstly, the open reading frames (ORFs) and their corresponding protein sequences were identified from the transcripts of the transcriptome using the TransDecoder tool in the Trinity pipeline. Identification of the unigenes (unique ORFs) was carried out using CD-HIT-EST (v4.6.8) with an identity threshold of 99%, a word length of 10 (the size of the sequence used in each cycle of comparison), and with the mode to cluster the most similar sequences [4]. The transcriptome was annotated using the BLASTx and BLASTp tools [5]. NCBI non-redundant protein database [6], UniProt [7], and Pfam [8] were used to search for protein homologs in the ORFs with a cut-off e-value of 1e-5. InterProScan (v5.32-71.0) was used to scan the ORFs for various protein signatures [9]. Databases, namely CATH-Gene3D, CDD, HAMAP, MobiDB, PANTHER, Pfam, PIRSF, PRINTS, ProDom, PROSITE, SMART, SUPERFAMILY, SFLD, and TIGRFAMs were used to identify domains or motifs of proteins represented by the ORFs through InterProScan. GO terms assigned by InterProScan were used by WEGO (v2.0) for the GO annotation of ORFs [10].

KEGG (Kyoto Encyclopedia of Genes and Genomes), GhostKOALA (KEGG orthology and links annotation), and KEGG automated annotation service (KAAS) were used to assign KEGG-orthology (KO annotation) to ORFs. Among the KO annotations, KAAS was given precedence as it identified KO based on a comparison with related species, whereas the GhostKOALA searches the prokaryotic, eukaryotic, and viral sequences. The amino acid unigene sequences were BLASTed against the genes of selected organisms. In our case, we BLASTed against plants including *Brassica napus* (rape), *Citrus sinensis* (Valencia orange), *Theobroma cacao* (cacao), *Gossypium raimondii*, *Gossypium hirsutum* (upland cotton), *Glycine max* (soybean), *Medicago truncatula* (barrel medic), *Rosa chinensis* (China rose), *Vitis vinifera* (wine grape), *Solanum lycopersicum* (tomato), *Nicotiana tabacum* (common tobacco), *Olea europaea* var. sylvestris (wild olive), *Helianthus annuus* (common sunflower), *Oryza sativa* japonica (Japanese rice) (RefSeq), and *Zea mays* (maize) based on the NR BLASTx search results of the total ORFs. A few mammals including *Rattus norvegicus* (rat), *Bos taurus* (cow) and *Homo sapiens* (human), fish *Danio rerio* (zebrafish), insect *Drosophila melanogaster* (fruit fly), and nematode *Caenorhabditis elegans* were considered because they are well studied in the genetic aspect and contain the steroid biosynthesis pathway genes which are of central interest in the current study. A few fungi, such as *Saccharomyces cerevisiae* (budding yeast), *Ashbya gossypii* (Eremothecium gossypii), *Candida albicans*, *Schizosaccharomyces pombe* (fission yeast), and *Encephalitozoon cuniculi* were included to represent majority groups of fungi. Protists such as *Entamoeba histolytica*, *Plasmodium falciparum* 3D7, and *Cryptosporidium hominis*, and prokaryotes such as *Escherichia coli* K-12 MG1655, *Neisseria meningitidis* MC58 (serogroup B), *Helicobacter pylori* 26695,

Bacillus subtilis subsp. subtilis 168, *Lactococcus lactis* subsp. lactis Il1403, *Mycoplasma genitalium* G37, *Mycobacterium tuberculosis* H37Rv, *Synechocystis* sp. PCC 6803, *Aquifex aeolicus*, *Methanocaldococcus jannaschii*, and *Aeropyrum pernix* were used for the same reason as mentioned above. A total of 859,009 KEGG sequences were used in the bi-directional best hit method of KAAS BLAST functional annotation of unigenes.

The Reconstruct Pathway tool of KEGG was used to process the KO annotations and further classify the ORFs based on KEGG metabolic pathways, BRITE (hierarchical classifications of biological entities), and modules (functional units of pathways).

Identification of differentially expressed transcripts

Trinity pipeline was used for differential expression (DE) analysis. As a prerequisite to DE analysis, abundance estimation of the transcripts was done using the alignment-based abundance estimation method, namely "RNA-Seq by Expectation-Maximization" (RSEM) [11]. The abundance data of all the 317,983 transcripts were represented as transcripts per million (TPM) and fragments per kilobase of transcript per million mapped reads (FPKM). A raw counts matrix and a normalized expression matrix were then generated, containing expression data for each replicate of each sample. A "trimmed mean of M values" (TMM) normalization method was used to do cross-sample normalization [12]. Further, the Trinity script using edgeR, a Bioconductor package, was used to conduct the DE analysis to obtain $\log_2$FPKM, log fold change (logFC), log counts per million (logCPM), p-value, and false discovery rate (FDR) for each transcript. Finally, the expression level of each transcript was studied as TPM where the normalization is first done for transcript length and then for sequence depth to obtain an expression unit that can span different samples and replicates of experiment [13].

Further, the expression of selected transcripts was studied by plotting the expression data as a hierarchal tree using Multiple Experiment Viewer (v4.9). Thus, the expression patterns of the various transcripts in relation to each other were observed, and co-expressed transcripts were studied as clusters.

Identification of transcription factor and protein kinase families

The iTAK (v1.7a) stand-alone tool was used to identify plant transcription factors (TFs), transcription regulators (TRs), and protein kinases (PKs) from the protein sequences obtained from the transcriptome via Transdecoder. TFs, TRs, and PKs were further individually classified internally by iTAK to their respective gene families. The documentation and cataloging of the various TFs and TRs were based on the consensus rules drafted from the PlnTFDB database [14] and the PlantTFDB portal [15]. The database entries of iTAk are well-curated to ensure accuracy. Plant PKs are identified based on protein kinase domains (Pfam domains PF00069 and PF07714). The identified PK hits are further classified internally by iTAK using the respective protein kinase Hidden Markov Models (HMMs) [16].

Identification of simple sequence repeats (SSRs)

The SSR motifs were identified among the transcripts of the leaf and root transcriptome using the MISA stand-alone PERL tool [17]. The parameters used to define the microsatellites were a minimum of 6 repeated for a unit size of 2 nt, a minimum of four repeats for a unit size of 6 nt, a minimum of five repeated for the unit sizes of 3 nt, 4 nt and 5 nt. Mononucleotide motifs were not considered in the current analysis due to the chance of homopolymer tail artifacts formed

during sequencing. Simple repeat motifs comprising one SSR and compound repeat motifs containing two or more SSRs with a maximum of 100 nt interrupting them were considered.

BUSCO analysis

Completeness of the transcriptome was assessed using BUSCO[18]. Analysis was performed in transcriptome mode using the eudicots_odb10 database.

Gene isolation and cloning

pEAQ for Nicotiana benthamiana expression. Genes of interest were PCR-amplified from the cDNA of *D. lanata* leaves using Platinum™ Taq (Invitrogen, Waltham, MA, USA) or Phusion® DNA polymerase (New England Biolabs (NEB), Ipswitch, MA, USA) and cloned into the AgeI and XhoI sites in the pEAQ-HT binary vector [19] by Gibson cloning [20]. Genes inserted were verified by Sanger sequencing (Genewiz, South Plainfield, New Jersey, USA).

MoClo system for yeast expression. We used the Golden Gate cloning based yeast MoClo system to clone genes of interest for yeast expression [21,22]. *D. lanata* $P450_{scc}$ was PCR-amplified from the pEAQ-HT_*Dl*$P450_{scc}$. Codon-optimized human $P450_{scc}$, along with its redox partners, *ADX* and *ADR*, were amplified from three pYTK001 part plasmids carrying these three genes. The *Arabidopsis* ATR2 gene codon-optimized for yeast expression was synthesized by TWIST biosciences (San Francisco, CA, USA). All genes were cloned into the MoClo pYTK001 entry vector using the Esp3I (a high-fidelity analog of BsmBI) restriction enzyme (NEB, Ipswitch, MA, USA). The following point mutations were made in *Dl*CYP87A4 using primers specific for S123A, A355V, and L357A mutations. Genes cloned were verified by Sanger sequencing (Genewiz, South Plainfield, New Jersey, USA). Transcription-unit (TU) plasmids were assembled with promoters and terminators from the MoClo kit using the high-fidelity BsaI restriction enzyme (NEB, Ipswitch, MA, USA). The transcription units were then used to assemble a multigene plasmid with a 2μ yeast origin of replication.

Gas chromatography coupled mass spectrometry (GC/MS) analysis for cholesterol and phytosterol quantification.

For steroid analysis, *D. lanata* leaf and root samples were prepared as described previously by Itkin, M. et. Al [23]. Yeast samples from three biological repeats were prepared by lysing OD_{600}=1 of cells. Yeast cells were pelleted at 500g for 4 minutes, resuspended in 200 μL of TES buffer, and homogenized with an equal volume of 0.5 mm glass beads in a BBX24 Bullet Blender® homogenizer (Next Advance, Troy, NY, USA) at setting 8 at 4˚C for 4 minutes. 300 μL of TES buffer was added to the lysed cells, and 400-500 μL of the yeast lysate was transferred into a capped glass tube and resuspended in 6 mL chloroform/methanol mix (2:1, v/v). Samples were heated at 75˚C for 60 min. The solvent was dried under a stream of air and resuspended in 2 mL of 6% (w/v) KOH in 100% methanol. Samples were saponified by heating at 90 ˚C for 60 minutes. They were then cooled to room temperature and 1.5 mL hexane, and 1.5 mL ddH_2O were added, followed by vigorously shaken by hand for 20 sec. The organic hexane phase was separated by centrifugation at 3,000g for 2 min, and 1 mL was transferred to a new glass test tube. The sample was dried under a stream of air and resuspended in 50μL of N-methyl-N-(trimethylsilyl) trifuoroacetamide (MSTFA) (Fisher Scientific, Waltham, MA, USA), vortexed to mix for 20 sec, and transferred to a glass insert inside a GC glass vial for derivatization.

Samples were analyzed on the Thermo Scientific™ Q-Exactive™ GC hybrid quadrupole Orbitrap™ (Fisher Scientific, Waltham, MA, USA). Samples were injected into a Thermo Scientific TraceGOLD TG-5SILMS column (Catalog number: 26096-2760) that was 30 m long, had a 0.2 mm inner diameter and 0.25 film thickness. The inlet temperature was set at 280°C. The splitless injection volume was 5 µL. Helium was used as carrier gas at a flow rate of 1.2 mL/min. The thermal gradient started at 170°C and held for 1.5 min, then ramped to 280°C at 37°C/min, further ramped to 300°C at 1.5°C/min, and finally held at 300°C for 5.0 min. Eluents were ionized by electron impact ionization (EI) at 70 eV. A filament delay of 6 min was used. High-resolution EI fragment spectra were acquired in the full scan mode using 60,000 resolution with a mass range of 50-500 m/z. The scan rate was set at automatic to ensure sufficient ions hitting the detector. The mass spectrometer was regularly calibrated to ensure mass accuracy. Quantification of sterols was performed using the Xcalibur™ software v4.4.16.14 based on standard curves built using pure sterol standards with concentrations ranging from 0.78 mg/L to 50 mg/L. The ions of observed m/z 329.3202 and 368.3436 were used for quantifying cholesterol, m/z 343.3359 and 382.3593 for campesterol, m/z 379.3358 and 394.3592 for stigmasterol, and m/z 357.3515 and 396.3750 for b-sitosterol. The Δ m/z is 5 ppm. Three biological replicates were used for quantification in each sample.

Real-time polymerase chain reaction (RT-PCR)

Leaves from 3-4 weeks old *D. lanata* seedlings were used for RNA isolation. Freshly collected samples in biological triplicates were snap-frozen in liquid nitrogen and ground into powder. Trizol (Ambion, Austin, TX, USA) reagent was used to isolate total RNA according to the manufacture's protocol. DNA in the sample was removed using the TURBO DNA-*free*™ kit (Invitrogen, Waltham, MA, USA). cDNA was synthesized using the iScript™ cDNA Synthesis Kit (BIO-RAD, Hercules, CA, USA) as per the manufacturer's instructions. qRT-PCR was carried out using the iTaq Universal SYBR Green Supermix (BIO-RAD, Herculese, CA, USA) with *D. lanata* cDNA, gene-specific primers. The following thermocycling conditions were set on CFX Maestro (v 4.1.2433.1219) software and were; denaturation at 95 °C for 3 min, 40 cycles of denaturation at 95 °C for 10 sec, and annealing at 55 °C for 30 sec. A melting curve was generated by increasing the temperature from 55 °C to 95 °C in 0.5 °C increments for 81 cycles. Three biological replicates and two technical replicates were included for each sample. Polyubiquitin 10 (UBQ10) was used as an internal standard. Primers are listed in Supplementary Table S3.

Phylogenetic analysis

Cytochrome P450s for the cytochrome P450 tree in Supplementary Figure 7 were retrieved by searching the D. lanata transcriptome for transcripts that encoded proteins containing the Pfam domain PF00067 (CYPs) and were between 400-600 amino acids in length. In addition, Arabidopsis thaliana sequences were retrieved from the Arabidopsis CYP database [24].

Supplementary Notes

Supplementary Note 1. Transcriptome sequencing and its de novo assembly

The transcriptome sequencing of leaf and root samples provided a total of 173,448,870 raw reads with an average length of 100 bp. After quality assessment using FastQC and read trimming by Trimmomatic, 173,445,956 high-quality reads were used for transcriptome assembly. A gentler trimming strategy (PHRED = 5) was employed to remove only the lowest-quality bases, thus retaining the shorter and lesser expressed transcripts, which were vulnerable to lose in case of a harsh trimming [25]. A total of 310,473,283 bp were assembled into 317,983 transcripts by the Trinity transcriptome assembler containing 183,152 Trinity genes (distinct groups of transcripts identified by Trinity assembler which contain sequences greatly similar to each other and are considered as isoforms). The average size of transcripts was 976 bp for the entire transcriptome. The size distribution of the total assembled transcripts in the transcriptome is consistent with already existing plant transcriptomes such as *Salvia miltiorrhiza* [26], *Withania somnifera* [27] , *Chrysanthemum morifolium* [28], *Calotropis procera* [29], *Persea americana* Mill. (Avocado), *Macadamia integrifolia* L. (macadamia), and *Mangifera indica* L. (mango) [30] (Figure S1A). The large number of transcripts whose sizes were greater than 3,500 bp was attributed to the overzealous production of long isoforms by the Trinity assembler (Figure 1A).

Raw reads mapped onto the transcriptome assembly using Bowtie2 (v2.3.4.3) showed 99.36% alignment indicating an excellent quality of the assembled transcriptome [31]. The transcriptome had an overall N50 value of 1,712 bp. Plotting the ExN50 value against varying levels of cumulative transcript expression (Ex) identified a saturation point of the assembly at 88% of the total expression, giving an improved E88N50 of 1,990 bp and reducing the effective transcripts, or transcripts contributing to the saturation point, count to 70,502 (Figure 1B). According to Trinity, the ExN50 peak begins to shift towards ~90% as the read depth increases. Therefore, in this case, since the ExN50 peaks at 88%, the read depth is considered sufficient. This suggests that the assembled transcriptome has a saturation of full-length reconstructed transcripts on account of its read depth (https://github.com/trinityrnaseq/trinityrnaseq/wiki/Transcriptome-Contig-Nx-and-ExN50-stats).

Supplementary Note 2. Transcriptome annotation

Multiple open reading frames (ORFs) were observed in most of the transcripts in the transcriptome, but a good number of them were less than 100 amino acids (300 bp), likely resulting from artifacts of assembly. A total of 190,755 ORFs were obtained from the transcripts using the “TransDecoder.LongOrfs” tool; all the ORFs thus found presented a minimum of 100 amino acids (default for the TransDecoder.LongOrfs tool), among them 121,298 (63.59%) had a methionine start codon and a stop codon, therefore considered complete.

The total number of 5’ partials (33,774), or ORFs with a start codon but no stop codon, were a little more than double of the 3’ partials (15,036), this is usually seen if poly(A) enrichment is used in the library preparation process [32], but in our case, we used the TrueSeq Ribo-Zero Plant RNA library prep kit with Ribo-Zero ribosomal RNA reduction chemistry and still found similar results. Also, 20,647 sequences were considered internal since they were both 5’ and 3’ partials. The 190,755 ORFs were annotated with NCBI non-redundant protein database (NR), and 161,326 of the total ORFs were annotated using BLASTx. Taxonomic analysis of the BLAST results revealed that 84.57% of the ORFs matched previously annotated genes, out of which 86.71% represent eukaryotic genes; among the eukaryotic genes, 92.64% represent plant genes.

About 12.88% of the total annotated ORFs represent bacterial genes, and only 0.39% represent archaea. Prominent plant species whose genes are homologous to the annotated ORFs are *Sesamum indicum* (Pedaliaceae), *Olea europaea* (Oleaceae), *Erythranthe guttata* (Phrymaceae), and *Handroanthus impetiginosus* (Bignoniaceae), all belonging to the order of Lamiales, of which *Sesamum indicum* showed a maximum coverage of 75.26% (Figure 1C). UniProt non-redundant curated proteins database (Swiss-Prot) annotated 92,133 (48.30%) ORFs, among which 79,137 (85.89%) were against plant genes. Among the plants, *Arabidopsis thaliana* rendered a maximum annotation of 61,617 (66.88%) ORFs, followed by *Oryza sativa* (5.36%), *Nicotiana tabacum* (2.29%), *Solanum lycopersicum* (1.17%), and *Solanum tuberosum* (0.94%) (Figure 1D).

CD-HIT (v4.6.8) was used to identify 113,221 unigenes with an identity threshold of 99% out of the 190,755 ORFs. Most of the sequences lost are repeats of the same sequences, either complete or partial. The higher threshold of 99% was used so that alleles that have considerable (>1%) sequence variation are retained.

Supplementary Note 3. Gene ontology (GO) classification

Among the total unigenes, 42,724 (37.7%) were linked to gene ontology (GO) terms. 42,724 (44.2%) of these unigenes were classified in the biological process, 11,458 (10.1%) were in the cellular component, and 36,574 (32.3%) were in the molecular function category (Figure S2A). Unigenes in the biological process primarily belong to various metabolic processes, including cellular, organic substance, primary, and nitrogen compound metabolic processes. The majority of unigenes in the cellular component class belonged primarily to the cell part, intracellular part, organelle, and membrane subcategories. Unigenes in the molecular function group were primarily classified in the binding, catalytic activity, heterocyclic compound binding, and ion binding subgroups. A total of 379 GO accessions were observed in the overall GO classification.

Supplementary Note 4. Functional characterization using KEGG

The reconstruct pathway tool of the KEGG mapper analysis annotated 5,683 unigenes [33]. Among them were 3,922 KEGG orthologs (KO) and 1,983 enzymes. Genes and proteins were also identified under the general categories of metabolism, genetic information processing, and signal and cellular processing (Figure 2B). This was completed based on KEGG pathway maps, BRITE hierarchies, and KEGG modules.

Although KEGG mapping against BRITE hierarchies is a process similar to GO enrichment, KEGG BRITE currently has 53 classification systems, compared to only three in GO (Figure S3A)[33]. The KEGG mapper revealed representations of unigenes in 412 KEGG pathways, of which 84 modules were complete. Among the complete modules were the terpenoid backbone biosynthesis pathways, including the mevalonate pathway and the non-mevalonate pathway, as well as the mono-, sesqui-, and di-terpenoid biosynthetic pathways (Figure S3B). It also identified partial modules in sterol biosynthesis, such as cholesterol biosynthesis (8 out of 10 enzymes) (Figure S3B). Overall, there were 93 enzymes identified in the metabolism of terpenoids and polyketides.

Supplementary Note 5. Protein kinase, transcription regulator, and transcription factor families

Protein kinases (PKs), transcription regulators (TRs), and transcription factors (TFs) play a key role in plant development and response to environmental stimuli. Much is unknown, especially in the context of secondary metabolism, regarding the role of PKs, TRs, and TFs. Since *D. lanata* has a unique cardenolide pathway, identifying its PKs, TRs, and TFs would lay the foundation for further studies to understand the molecular mechanism of how stresses and environmental stimuli induce the cardenolide pathway.

In the *D. lanata* transcriptome, 126 PK sub-families were identified, of which the RLK-Pelle_DLSV PK sub-family had a maximum representation of 282 unigenes. Also, the RLK-Pelle family seems to be the major family of PK in *D. lanata,* many of the proteins in this family are receptor-like kinases, and the rest are cytoplasmic kinases that lost their extracellular domains [34]. A list of all the unigenes representing various PKs is found in Supplementary Data 4.

1,883 unigenes were identified as TRs*,* of which 201 unigenes were in the SNF2 (Figure S4A). The SNF2 family plays essential roles in cellular processes such as transcriptional regulation, maintenance of chromosome stability during mitosis, and DNA damage repair [35]. 5,634 unigenes are assigned to 66 TF families, of which the top representation was the bHLH family represented by 421 unigenes (Figure S4B). The basic helix-loop-helix (bHLH) is the most general class of TFs in eukaryotes. bHLH is commonly involved in plant growth and metabolism, especially in photomorphogenesis, light signal transduction, and secondary metabolism. It also plays a vital role in stress response [36]. A list of all the unigenes representing various TRs and TFs is available in Supplementary Data 5.

Supplementary Note 6. Simple sequence repeats (SSRs)

De novo assembled transcriptomes have proven to be a useful tool for molecular marker development [37]. Such markers are predominantly simple sequence repeats (SSRs), also called microsatellites. SSR markers identified from a transcriptome are termed expressed sequence tag-simple sequence repeat (EST-SSR) markers. Although usually low levels of polymorphism are detected in EST-SSRs compared with genomic SSRs, EST-SSRs can be successfully used for various purposes, and they may prove superior to genomic SSR markers for diversity estimation and transferability [38]. These EST-SSRs play a vital role in genetic diversity analysis and plant identification. These SSRs are short repeat sequences with unit sizes ranging from 1 nt to 7 nt. They may also contain more than one motif with overlapping nucleotides. A total of 22,549 SSRs were identified in the *D. lanata* transcriptome. The number of transcripts containing more than one SSR was 2,520, and the number found in compound formations was 1,520. Among the 2-nt, 3-nt, 4-nt, 5-nt, and 6-nt SSR motifs, the total 2-nt SSRs were predominant (12,530), followed by 3-nt SSRs (7,490). The lowest was the 4-nt (673). 5-nt (912), and 6-nt (944) motifs were almost equal. Most 2-nt SSR motifs were in the AG/CT, AT/AT, and AC/GT categories. The 3-nt SSR motifs were predominantly AAG/CTT, AAT/ATT, ATC/ATG, ACC/GGT, AGC/CTG, AGG/CCT, AAC/GTT, ACT/AGT, CCG/CGG, and ACG/CGT. The 4-nt SSR motifs were mostly ACAT/ATGT, AAAT/ATTT, and AAAG/CTTT, and the 5-nt SSR motifs were represented by AAAAT/ATTTT, AAACC/GGTTT, AAAAG/CTTTT, and AAAAC/GTTTT. The 6-nt SSRs motifs were a minority and, therefore, not shown in Table 1). The output of the MISA tool used to identify the SSRs from the transcriptome and the statistical data are available in Supplementary Data 6 and Supplementary Data 7 respectively.

Supplementary References

1 Andrews, S. *FastQC: a quality control tool for high throughput sequence data*, <http://www.bioinformatics.babraham.ac.uk/projects/fastqc> (2010).

2 Haas, B. J. *et al. De novo* transcript sequence reconstruction from RNA-seq using the Trinity platform for reference generation and analysis. *Nature protocols* **8**, 1494 (2013).

3 Langmead, B. & Salzberg, S. L. Fast gapped-read alignment with Bowtie 2. *Nature methods* **9**, 357-359 (2012).

4 Li, W. & Godzik, A. Cd-hit: a fast program for clustering and comparing large sets of protein or nucleotide sequences. *Bioinformatics* **22**, 1658-1659 (2006).

5 Altschul, S. F., Gish, W., Miller, W., Myers, E. W. & Lipman, D. J. Basic local alignment search tool. *Journal of molecular biology* **215**, 403-410 (1990).

6 Pruitt, K. D., Tatusova, T. & Maglott, D. R. NCBI reference sequences (RefSeq): a curated non-redundant sequence database of genomes, transcripts and proteins. *Nucleic acids research* **35**, D61-D65 (2007).

7 Consortium, U. The universal protein resource (UniProt) in 2010. *Nucleic acids research* **38**, D142-D148 (2010).

8 Finn, R. D. *et al.* Pfam: the protein families database. *Nucleic acids research* **42**, D222-D230 (2014).

9 Quevillon, E. *et al.* InterProScan: protein domains identifier. *Nucleic acids research* **33**, W116-W120 (2005).

10 Ye, J. *et al.* WEGO 2.0: a web tool for analyzing and plotting GO annotations, 2018 update. *Nucleic acids research* **46**, W71-W75 (2018).

11 Li, B. & Dewey, C. N. RSEM: accurate transcript quantification from RNA-Seq data with or without a reference genome. *BMC bioinformatics* **12**, 323 (2011).

12 Robinson, M. D. & Oshlack, A. A scaling normalization method for differential expression analysis of RNA-seq data. *Genome biology* **11**, R25 (2010).

13 Wagner, G. P., Kin, K. & Lynch, V. J. Measurement of mRNA abundance using RNA-seq data: RPKM measure is inconsistent among samples. *Theory in biosciences* **131**, 281-285 (2012).

14 Pérez-Rodríguez, P. *et al.* PlnTFDB: updated content and new features of the plant transcription factor database. *Nucleic acids research* **38**, D822-D827 (2010).

15 Jin, J., Zhang, H., Kong, L., Gao, G. & Luo, J. PlantTFDB 3.0: a portal for the functional and evolutionary study of plant transcription factors. *Nucleic acids research* **42**, D1182-D1187 (2014).

16 Lehti-Shiu, M. D. & Shiu, S.-H. Diversity, classification and function of the plant protein kinase superfamily. *Philosophical Transactions of the Royal Society B: Biological Sciences* **367**, 2619-2639 (2012).

17 Thiel, T., Michalek, W., Varshney, R. & Graner, A. Exploiting EST databases for the development and characterization of gene-derived SSR-markers in barley (*Hordeum vulgare* L.). *Theoretical and applied genetics* **106**, 411-422 (2003).

18 Manni, M., Berkeley, M. R., Seppey, M., Simão, F. A. & Zdobnov, E. M. BUSCO update: novel and streamlined workflows along with broader and deeper phylogenetic coverage for scoring of eukaryotic, prokaryotic, and viral genomes. *Molecular Biology and Evolution* **38**, 4647-4654 (2021).

19 Sainsbury, F., Thuenemann, E. C. & Lomonossoff, G. P. pEAQ: versatile expression vectors for easy and quick transient expression of heterologous proteins in plants. *Plant biotechnology journal* **7**, 682-693 (2009).

20 Gibson, D. G. *et al.* Enzymatic assembly of DNA molecules up to several hundred kilobases. *Nature methods* **6**, 343-345 (2009).

21 Lee, M. E., DeLoache, W. C., Cervantes, B. & Dueber, J. E. A highly characterized yeast toolkit for modular, multipart assembly. *ACS synthetic biology* **4**, 975-986 (2015).

22 Mukherjee, M., Caroll, E. & Wang, Z. Q. Rapid assembly of multi-gene constructs using modular golden gate cloning. *J Vis Exp* **168**, e61993 (2021).

23 Itkin, M. *et al.* GLYCOALKALOID METABOLISM1 is required for steroidal alkaloid glycosylation and prevention of phytotoxicity in tomato. *The Plant Cell* **23**, 4507-4525 (2011).

24 Paquette, S. M., Bak, S. & Feyereisen, R. Intron–exon organization and phylogeny in a large superfamily, the paralogous cytochrome P450 genes of *Arabidopsis thaliana*. *DNA and cell biology* **19**, 307-317 (2000).

25 MacManes, M. D. On the optimal trimming of high-throughput mRNA sequence data. *Frontiers in genetics* **5**, 13 (2014).

26 Yang, L. *et al.* Transcriptome analysis of medicinal plant *Salvia miltiorrhiza* and identification of genes related to tanshinone biosynthesis. *PLoS One* **8** (11), e80464 (2013).

27 Gupta, P. *et al. De novo* assembly, functional annotation and comparative analysis of *Withania somnifera* leaf and root transcriptomes to identify putative genes involved in the withanolides biosynthesis. *PLoS One* **8** (5), e62714 (2013).

28 Wu, Y.-H. *et al.* Comparative analysis of the chrysanthemum leaf transcript profiling in response to salt stress. *PloS one* **11** (7), e0159721 (2016).

29 Pandey, A. *et al.* Transcriptome and metabolite analysis reveal candidate genes of the cardiac glycoside biosynthetic pathway from *Calotropis procera*. *Scientific reports* **6**, 34464 (2016).

30 Chabikwa, T. G., Barbier, F. F., Tanurdzic, M. & Beveridge, C. A. *De novo* transcriptome assembly and annotation for gene discovery in avocado, macadamia and mango. *Scientific Data* **7**, 1-7 (2020).

31 Langmead, B., Trapnell, C., Pop, M. & Salzberg, S. L. Ultrafast and memory-efficient alignment of short DNA sequences to the human genome. *Genome biology* **10**, R25 (2009).

32 Wang, Z., Gerstein, M. & Snyder, M. RNA-Seq: a revolutionary tool for transcriptomics. *Nature reviews genetics* **10**, 57-63 (2009).

33 Kanehisa, M. & Sato, Y. KEGG Mapper for inferring cellular functions from protein sequences. *Protein Science* **29**(1), 28-35 (2019).

34 Gish, L. A. & Clark, S. E. The RLK/Pelle family of kinases. *The Plant Journal* **66**, 117-127 (2011).

35 Eisen, J. A., Sweder, K. S. & Hanawalt, P. C. Evolution of the SNF2 family of proteins: subfamilies with distinct sequences and functions. *Nucleic acids research* **23**, 2715-2723 (1995).

36 Sun, X., Wang, Y. & Sui, N. Transcriptional regulation of bHLH during plant response to stress. *Biochemical and biophysical research communications* **503**, 397-401 (2018).

37 Chen, H. *et al.* Transcriptome sequencing of mung bean (*Vigna radiate* L.) genes and the identification of EST-SSR markers. *PloS one* **10** (4), e0120273 (2015).

38 Gupta, P. K. *et al.* Transferable EST-SSR markers for the study of polymorphism and genetic diversity in bread wheat. *Molecular genetics and genomics* **270**, 315-323 (2003).

39 Souza, C. M. *et al.* A stable yeast strain efficiently producing cholesterol instead of ergosterol is functional for tryptophan uptake, but not weak organic acid resistance. *Metabolic engineering* **13**, 555-569 (2011).
