# Supplementary material for: A cytochrome P450 CYP87A4 imparts sterol side-chain cleavage in digoxin biosynthesis
Source: Nat Commun. 2023 Jul 8;14:4042. doi: 10.1038/s41467-023-39719-4 (PMC10329713; doi:10.1038/s41467-023-39719-4)
Supplement: Supplementary file 3 — Description of Additional Supplementary Files [file 41467_2023_39719_MOESM3_ESM.pdf]

Description of Additional Supplementary Files:

Supplementary Data 1. *D. lanata CYP87A1-4* nucleotide sequences

Supplementary Data 2. *D. lanata* CYP87A1-4 amino acid sequences

Supplementary Data 3. Codon-optimised sequences

Supplementary Data 4. List of all the unigenes representing various PKs

Supplementary Data 5. List of all the unigenes representing various TRs and TFs

Supplementary Data 6. Output of the MISA tool used to identify the SSRs from the transcriptome

Supplementary Data 7. Summary of output of the MISA tool used to identify the SSRs from the transcriptome
